# Oscillations of the Wandering Mind: Neural Evidence for Distinct Exploration/Exploitation Strategies in Younger and Older Adults

**DOI:** 10.1002/hbm.70174

**Published:** 2025-04-27

**Authors:** Catherine N. Moran, David P. McGovern, Mike Melnychuk, Alan F. Smeaton, Paul M. Dockree

**Affiliations:** ^1^ Trinity College Institute of Neuroscience & School of Psychology Trinity College Dublin Dublin Ireland; ^2^ School of Population Health RCSI University of Medicine & Health Sciences Dublin Ireland; ^3^ School of Psychology Dublin City University Dublin Ireland; ^4^ Insight Centre for Data Analytics Dublin City University Dublin Ireland

**Keywords:** ageing, electroencephalography, exploitation/exploration, mind‐wandering, oscillatory dynamics, pupillometry, sustained attention

## Abstract

This study traced the neurophysiological signals of fluctuating attention and task‐related processing to ascertain the mechanistic basis of transient strategic shifts between competing task focus and mind‐wandering, as expressed by the ‘exploitation/exploration’ framework, and explored how they are differentially affected with age. Thirty‐four younger (16 female, mean age 22 years) and 34 healthy older (20 female, mean age 71 years) adults performed the Gradual Contrast Change Detection task; monitoring a continuously presented flickering annulus for intermittent gradual contrast reductions and responding to experience sampling probes to discriminate the nature of their thoughts at discrete moments. Electroencephalography and pupillometry were concurrently recorded during target‐ and probe‐related intervals. Older adults tracked the downward stimulus trajectory with greater sensory integrity (reduced target SSVEP amplitude) and demonstrated earlier initiation of evidence accumulation (earlier onset CPP), attenuated variability in the attentional signal (posterior alpha) and more robust phasic pupillary responses to the target, suggesting steadier attentional engagement with age. Younger adults only exhibited intermittent sensory encoding, indexed by greater variability in the sensory (SSVEP) and attentional (alpha) signals before mind‐wandering relative to focused states. Attentional variability was accompanied by disrupted behavioural performance and reduced task‐related neural processing, independent of age group. Together, this elucidates distinct performance strategies employed by both groups. Older adults suspended mind‐wandering and implemented an exploitative oscillation strategy to circumvent their reduced cognitive resources and allay potential behavioural costs. Conversely, younger adults exhibited greater exploration through mind‐wandering, utilising their greater cognitive resources to flexibly alternate between competing goal‐directed and mind‐wandering strategies, with limited costs.

## Graphical abstract

This research reveals that older adults demonstrate steadier attentional engagement and earlier evidence accumulation, favouring a more exploitative task approach. In contrast, younger adults exhibit more frequent mind‐wandering and perceptual decoupling, flexibly shifting between exploration and exploitation without compromising overall task performance.

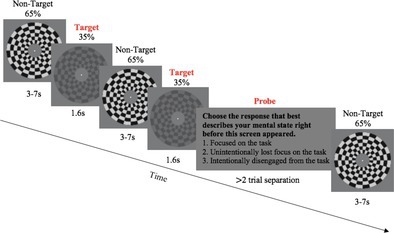

**Key Messages**

- *Age*‐*related stability in attentional engagement*: Our findings reveal that older adults exhibit steadier attentional engagement before target onset, as indicated by reduced variability in the attentional signal and a more consistent phasic pupillary response to targets. This suggests a shift towards a more exploitative and conservative task strategy with advancing age, which enhances performance stability by maintaining a more continuous focus on task‐relevant information.
- *Perceptual decoupling in younger adults during mind*‐*wandering*: Younger adults demonstrate greater perceptual decoupling during mind‐wandering, as evidenced by increased variability in sensory encoding and attentional signals during self‐reported mind‐wandering relative to focused states. This indicates a tendency to flexibly shift between focused and mind‐wandering states, leveraging their cognitive resources to explore without negatively impacting overall task performance. In contrast, older adults show a more consistent sensory representation, reflecting a more cautious and task‐oriented strategy.
- *Enhanced sensory encoding and decision*‐*making in older adults*: Older adults show an earlier onset of evidence accumulation and stronger sensory encoding of the target stimulus compared to younger adults. This pattern supports a more proactive and sustained approach to task engagement in older adults, who appear to prioritise accuracy and stability over exploratory behaviours, thereby mitigating potential performance costs associated with mind‐wandering and reduced cognitive resources.

## 1 Introduction

The *exploitation/exploration trade*‐*off* (Sripada 2018) posits an antagonistic alternation between ‘exploitative’ goal‐directed processes and ‘explorative’ modes of thinking, namely mind‐wandering. Goal‐directed thinking is understood as an exploitative process whereby available resources and known informational stores are utilised in the pursuit of a goal (Botvinick and Cohen 2014; Evans 2008). Mind‐wandering is an explorative state involving an open‐ended search for unknown but potentially more advantageous opportunities. Some preliminary studies have shown that mind‐wandering can, at times, be beneficial (Mooneyham and Schooler 2013). Some such benefits are conferred onto creative incubation and creative problem‐solving (e.g. Baird et al. 2012; Dijksterhuis and Meurs 2006, Gable et al. 2019; Leszczynski et al. 2017; Yamaoka and Yukawa 2020; although see contrary findings in Murray et al. 2024), memory encoding (Maillet and Schacter 2016; Maillet et al. 2017) and prospective memory and autobiographical planning (e.g. Baird et al. 2011; Girardeau et al. 2023). Optimal decision‐making and effective performance require balanced and flexible regulation between these exploit/explore strategies on a trial‐by‐trial basis in response to changing contextual demands or temporal uncertainties. Rather than a precise dichotomy, these serial modes of thought likely reflect a continuum, whereby actions appear comparatively more exploitative or exploratory, and more or less adaptive, relative to the context. Reaction time variability (RTV) has been proposed as a marker of such temporal oscillatory attention cycles (Esterman et al. 2013). RTV has been shown to fluctuate over time with periods of increased variability often associated with more frequent mind‐wandering and performance errors, and is more pronounced in younger adults (Cheyne et al. 2009; Esterman et al. 2013; Mata et al. 2013; McGovern et al. 2018; Moran et al. 2021; Seli et al. 2013). The exploitation/exploration framework when applied to cognitive ageing may, therefore, elucidate the role of different oscillatory dynamics in age‐related mind‐wandering patterns. However, the degree to which the natural ageing process interacts with this capacity for optimal strategic regulation, and the posited underlying mechanisms, remains largely unknown.

The locus–coeruleus noradrenaline (LC‐NA) neuromodulatory system, implicated in arousal, vigilance, and attentional control (Berridge and Waterhouse 2003; Coull et al. 2004; Sara and Bouret 2012; Smith and Nutt 1996), may represent one mechanism through which temporal oscillatory attentional cycles are regulated over time (O'Callaghan et al. 2021; Sripada 2018). Empirical evidence supports pupil diameter (PD) as a non‐invasive indirect proxy psychophysiological measure of LC‐NA activity, with non‐luminance‐mediated changes in PD tracking fluctuations in arousal, attentional allocation, and strategic shifts between exploitative and explorative cognitive states (Aston‐Jones and Cohen 2005; Joshi et al. 2016; Murphy et al. 2014). Within the adaptive gain framework, LC‐NA dynamics, as reflected in PD, govern the balance between focused task engagement and exploratory behaviour, following an inverted U‐shaped relationship with task performance (Aston‐Jones and Cohen 2005; van den Brink et al. 2016). With regard to ageing, research has proposed the roles of executive function and neuromodulatory influence in the navigation between competing exploit/explore strategies (Cohen et al. 2007; Hills et al. 2010; O'Callaghan et al. 2021). Age‐related decline in executive resources and age‐accompanied catecholaminergic deficits (Backman et al. 2000) may limit flexible shifting between exploitative and exploratory states in older adults. Indeed, age‐related differences in explorative tendencies have been previously documented (Mata et al. 2013; Moran et al. 2021). Moreover, greater neuronal density in the LC, indicative of more availability of NA, was observed in older adults who maintained better cognitive status with advancing age and showed attenuated pre‐mortem age‐related cognitive decline (Wilson et al. 2013). The exploit/explore framework, as indexed by LC‐NA activity and measured via PD, holds promise for delineating the patterns of changing performance and fluctuating subjective attentional states in younger and older adults over time.

This study aimed to investigate the extent to which younger and older adults strategically prioritise competing task‐relevant goals versus self‐generated thoughts during a non‐demanding, continuous sustained attention task. Against a backdrop of reduced executive function in older adults (Moran et al. 2021), we propose that younger and older adults employ different task‐related strategies to mitigate performance costs. Specifically, we hypothesise that older adults marshal their resources to the task by strategically consigning more of their limited resources to maintain an exploitative performance strategy and a more conservative decision policy (Sripada 2018). In contrast, we hypothesise that younger adults will permit greater shifts to exploratory states during the task. Here, we examine psychophysiological markers of (a) attentional fluctuations (EEG alpha and PD), (b) perceptual decoupling from task‐related sensory processing during mind wandering (steady‐state visually evoked potential [SSVEP]) and (c) perceptual decision‐making during goal‐directed behaviour (centro‐parietal positivity [CPP], SSVEP and left hemisphere beta [LHB]). Tracing key attentional, sensory and decision signals will help ascertain the mechanistic basis of strategic transient shifts in brain states, dually affected by competing sensory input and intrinsic processes, to augment our understanding of age‐related mind‐wandering.

## 2 Materials and Methods

### 2.1 Participants

Thirty‐five younger adults (aged 18–35 years) and 40 community‐dwelling healthy older adults (aged 65–80 years) participated in this study. Younger adults were recruited from the student population in Trinity College Dublin (TCD), while older adults were recruited from a research participant panel. All participants reported normal or corrected‐to‐normal vision, no personal or family history of neurological or psychiatric illness, no personal or family history of brain injury or unexplained fainting, no sensitivity to flickering light and no recent history of drug, alcohol or pharmaceutical abuse. Two younger participants were later excluded owing to technical data acquisition issues. Five older adults were excluded as they scored lower than 24 on the Montreal Cognitive Assessment (MoCA; Nasreddine et al. 2005), suggesting possible cognitive impairment (O'Caoimh et al. 2016) and one further older participant was excluded due to illness during testing.

Due to excessive electroencephalogram (EEG) and/or ocular artefacts, participants' *electroencephalographic* data were either removed from both the target‐ and probe‐related analyses (younger adults, *n* = 6; older adult, *n* = 1), the target analyses *only* (younger adults, *n* = 2; older adults, *n* = 3) or the probe analyses *only* (younger adults, *n* = 2; older adults, *n* = 3). Further, participants' *pupillometric* data were excluded from the target‐ *and* probe‐related analyses owing to excessive artefacts or missing data (younger adults, *n* = 1; older adults, *n* = 3). The sample sizes included in each analysis are detailed alongside each result.

The final sample of participants for whom we had data available for at least one of the neurophysiological analyses (EEG and/or pupillometry) comprised 34 younger adults (16 female, mean age 21.71 years, standard deviation [SD] = 4.59) and 34 older adults (20 female, mean age 70.97 years, SD = 3.54). The groups did not significantly differ with regard to sex, *x* ^2^(1, *N* = 68) = 0.94, *p* = 0.331, BF_10_ = 0.47, or years of full‐time education, *t*(60.99) = 1.47, *p* = 0.148 (two‐tailed), BF_10_ = 0.62. Younger adults reported an average of 16 years of education (SD = 2.41) and older adults reported 15 years (SD = 3.24). Participants were offered partial course credit (if students) or a €20 gratuity to cover travel costs. Participation was entirely voluntary, and all participants provided written informed consent to the procedures that were approved by the School of Psychology Research Ethics Committee, TCD and were conducted in observance of the Declaration of Helsinki principles and the European General Data Protection Regulations.

### 2.2 Experimental Design

The current study employed the gradual contrast change detection (Grad‐CCD) task with built‐in experience sampling (ES) probes (Grad‐CCD‐ES; McGovern et al. 2018; Moran et al. 2021; O'Connell et al. 2012) and concurrent EEG and pupillometry recordings to capture mind‐wandering under non‐demanding experimental conditions. These data were collected alongside a comprehensive neuropsychological test battery as part of a broader study that explored the neuropsychological factors mediating age‐related differences in mind‐wandering phenomenology. Results pertaining to the neuropsychological and behavioural analyses were previously disseminated (see Moran et al. 2021). Baseline neuropsychological findings revealed typical age‐related decrements on standardised tests of attention and executive function, consistent with existing research showing reduced cognitive resources with ageing (Braver and West 2008; Foster et al. 2007). Conversely, older adults reported experiencing less anxiety, less depression, and fewer daily‐life attentional difficulties compared to younger adults. Additionally, older adults demonstrated higher alertness before the task and reported less task‐induced motivation loss, supporting previous findings of enhanced positive affect and increased task interest in older populations (Jackson and Balota 2012; Krawietz et al. 2012; Parks Jr. et al. 1989).

The advantage of the present approach is the triangulation of subjective, behavioural, and neurophysiological methods. The paradigm is thus well‐suited to tracing and mechanistically dissociating the transitions between top‐down endogenous and bottom‐up stimulus‐evoked processes to explore how mind‐wandering impacts perceptual decision‐making and task performance over time and how these processes are affected differentially by ageing.

### 2.3 GradCCD‐ES Task Procedures

Healthy younger and older adult participants performed the GradCCD‐ES task in a darkened and sound‐attenuated room, sitting at a distance of ~57 cm from the presentation computer, with their heads supported by a chinrest to minimise head and eye movements. Participants monitored a continually presented annulus stimulus for smooth, gradually evolving, and temporally unpredictable feature changes over long and tedious blocks. This extended traditional methodological approaches that typically measure transient responses that are exogenously evoked by perceptually salient, sudden‐onset, discrete and often predictably occurring targets (e.g. a distinct symbol). The gradual stimulus changes in the present task minimised the need for rapid information processing and response demands, thereby placing greater reliance on endogenous attentional control and continued readiness and minimising the degree to which bottom‐up processes guided responding. The steady and gradually evolving target transitions removed momentary sensory‐evoked signals from the event‐related potential (ERP) and stimulus‐evoked pupillary responses, thereby enabling isolation of the individual dynamics of shifting attentional states as they occurred in real‐time. The visual annulus stimulus was presented in the centre display of a 40 cm cathode‐ray tube (CRT) monitor that operated at a 100 Hz refresh rate with 1024 × 768 resolution. Stimulus presentation and participant response collection regimes were controlled via the Psychtoolbox‐3 interface (Brainard 1997; Pelli 1997) and MATLAB R2016b software (MATLAB 2016). Participants fixated on the centre of the screen and monitored a continuously presented, flickering checkerboard annulus stimulus (outer radius = 8°, inner radius = 3°) with alternating light and dark radial segments on a dark grey background. The on–off flicker of the checkerboard stimulus at 25 Hz gave rise to an SSVEP in the EEG, which tracked the representation of the stimulus contrast, providing a neural read‐out of momentary sensory processing against which changes that occur with on and off‐task subjective states could be recorded (O'Connell et al. 2012).

**FIGURE 1:**
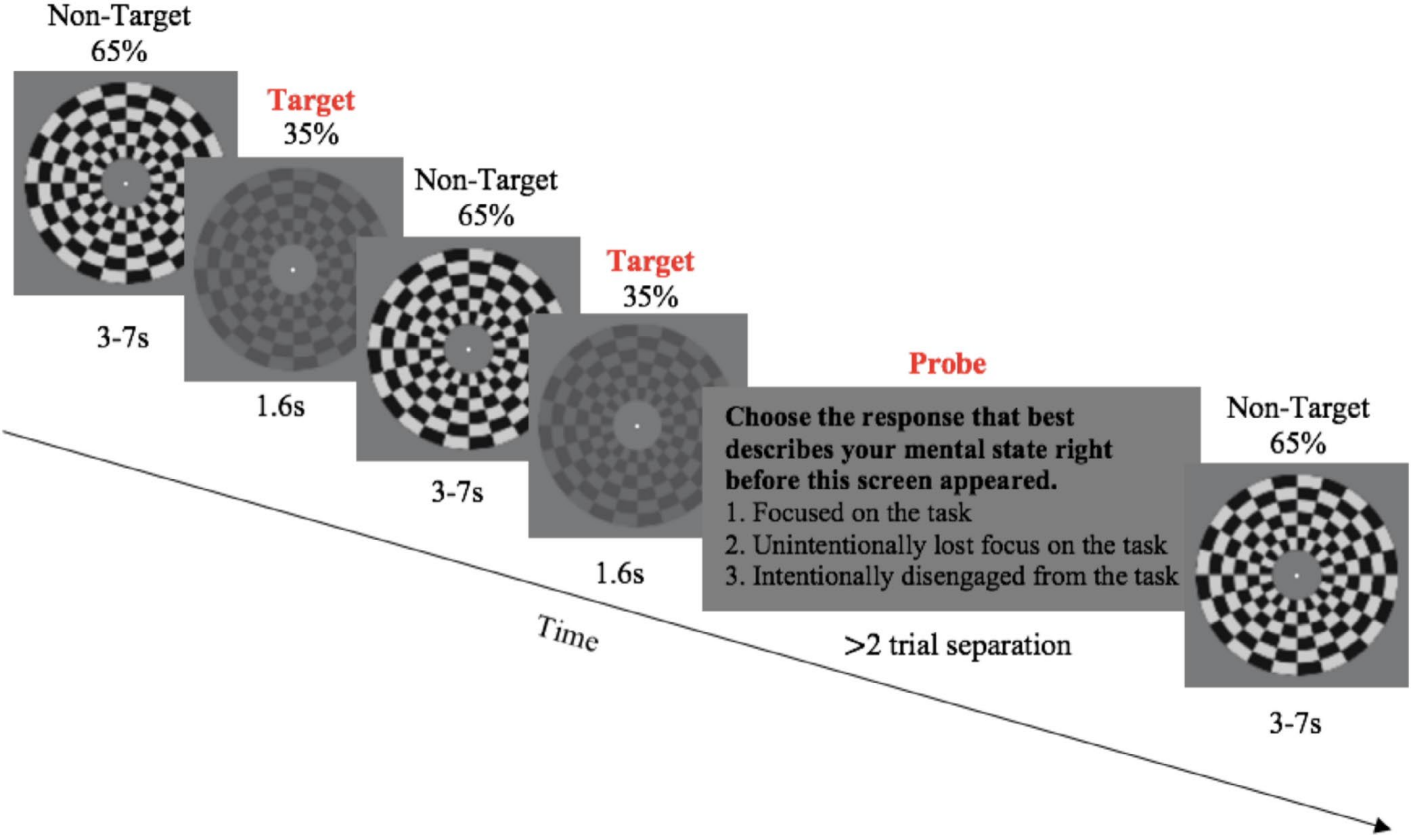
Experimental schematic depicting the gradual contrast change detection task with experience sampling probes (GradCCD‐ES). Participants continuously monitored the flickering annulus for gradual contrast changes, characterised as a stimulus contrast reduction from 65% to 35% over 1.6 s, and responded to intermittent experience sampling probes asking them about their current mental state. For the present report, unintentional and intentional mind‐wandering probe responses were collapsed to form a composite ‘Mind‐Wandering’ variable.

Participants identified intermittent targets, that took the form of gradual reductions in stimulus contrast from 65% to 35% over 1.6 s followed by a return to baseline after a further 0.8 s. Targets occurred periodically with inter‐trial intervals (ITIs) of three, five, or 7‐s selected randomly across trials to minimise target predictability and were long enough in duration to minimise possible phasic contamination of the pupil measurements from stimulus‐evoked pupil responses arising from previous trials. As soon as participants detected the target transition, they responded with a speeded mouse button press with their right index finger (Figure 1). The manual button press response to target identification enabled preparatory motor activity to be tracked over contralateral pre‐motor structures. The task was pseudo‐randomly interrupted by built‐in experience sampling (ES) probes (minimum two‐trial separation) that asked participants to classify their current mental state. Before the onset of each probe, the checkerboard stimulus offset was followed by a blank screen for 500 ms. The probe screen then instructed participants to ‘Choose the response that best describes your [their] mental state right before this screen appeared’. Participants indicated with a keyboard press (1–3) whether they had been (1) ‘Focused on the task’, (2) ‘Unintentionally lost focus on the task’ or (3) ‘Intentionally disengaged from the task’ in the moments before the onset of the probe (following Seli et al. 2015). Participants navigated their right hand to these keys to respond to the probe. Immediately following a probe response, the task resumed and participants returned their hands to rest on the mouse in expectation of possible targets. Before the main experiment, participants were provided task and probe instructions with mind‐wandering examples. Two brief practice blocks (including three trials and one probe) were administered to participants to ensure adequate understanding of the experimental procedures. Before each experimental block, the eye‐tracking system was calibrated and validated, and then the task was initiated on the display computer. Eight blocks of the main task were performed; each block contained 48 target trials and 16 probes and spanned approximately 8 min. Participants availed of short breaks in between blocks.

All behavioural performance and subjective ES indices were averaged across the total task for each participant. The behavioural outcomes extracted comprised (a) Hit Rate (proportion of correctly identified targets), (b) RT for correct trials (in seconds), (c) between‐trial RT coefficient of variance for correct trials (RT CoV; the standard deviation of RT divided by the mean RT) and (d) the number of False Alarms. Subjective outcomes included the frequency of (a) focused and (b) composite total mind‐wandering reports (sum of unintentional and intentional mind‐wandering responses). In the broader study (Moran et al. 2021), mind‐wandering experiences were dissociated as a means of classifying the phenomenology of mind‐wandering and examining age‐related patterns, and corollaries, of different types of self‐generated thought. The present report, however, investigated age‐related neurophysiological patterns of on‐ (focus) and off‐task (mind‐wandering) states, given that perceptual decoupling attributes no specific claims regarding intentionality. Moreover, concatenation of unintentional and intentional responses was performed to minimise data attrition in the neurophysiological analyses arising from low trial counts due to the infrequency of intentional mind‐wandering reports, particularly for older adults.

### 2.4 EEG Acquisition and Pre‐Processing

Continuous EEG was acquired by an ActiveTwo system (BioSemi, The Netherlands) using 128 scalp electrodes, according to the equiradial system montage, and digitised at 512 Hz. Vertical eye movements were recorded by two electrooculogram (EOG) electrodes positioned above and below the left eye. Data analyses were performed using custom scripts in MATLAB version r2016b (MATLAB 2016) incorporating EEGLAB functions for importing data files and spherical spline interpolation of noisy channels (Delorme and Makeig 2004). Continuous EEG data were detrended to suppress slow drifts and low‐pass filtered at 40 Hz using a zero‐phase non‐causal Hamming Windowed‐Sinc Finite Impulse Response (FIR) filter [‘eegfiltnew’ function] to mitigate high frequencies. Data were then re‐referenced offline to the average reference of all 128 electrodes. Data were segmented into fixed‐length stimulus‐locked epochs, according to the particular signal investigated and the time period of interest. Probe event triggers (i.e. probe presentation and response) were extracted from the behavioural files and converted to timestamps for use in the probe‐aligned analyses as these were not present in the electrophysiological outputs (.bdf files) due to a technical error.

For *target*‐*aligned analyses*, epochs were time‐locked to target onset using windows of either (a) −2000 ms to 200 ms (for pre‐target Alpha) or (b) −250 ms to 1925 ms (for decision‐related signals). The target‐aligned epochs were baseline corrected relative to the average signal in the 200 ms pre‐target window. Regarding the *probe*‐*aligned analyses*, data were segmented into epochs of −2000 ms before to 200 ms after probe presentation and baseline corrected relative to the −2000 to −1800 ms pre‐probe interval (pre‐probe SSVEP and Alpha).

Single ERP trials are sensitive to EOG or noise transients stemming from electrical interference from the recording environment, muscle activity, skin potentials, blinks and ocular movements. In the present study, trials containing artefacts were rejected if the bipolar vertical EOG signal (upper minus lower) exceeded an absolute value of 250 μV at any point during the epoch or if the scalp electrodes surpassed an artefact threshold of 100 μV. For probe‐aligned data, artefact rejection was restricted to the posterior channels (A‐lead) given that the signals of interest for the probe‐aligned analyses arise predominantly in the posterior region of the brain. To minimise trial loss during pre‐processing procedures, channels with extreme variance and/or high artefact counts were interpolated such that no more than 10% of channels were interpolated across the whole session for each participant. Following channel inspection and interpolation, participants with excessive trial loss, resulting in fewer than 30 trials remaining per variable, were removed from subsequent analyses (see Section 2.1). Moreover, for the probe‐related analyses, a further exclusion criterion was implemented such that participants with fewer than 10 total valid trials after pre‐processing procedures available for the focused (younger adult, *n* = 1) or mind‐wandering (younger adult, *n* = 1; older adults, *n* = 10) conditions were excluded from these analyses.

All single‐trial data were transformed into current source density (CSD; Kayser and Tenke 2006). This conversion was implemented to reduce spatial overlap between functionally distinct components, attenuate the spatial blurring effects of volume conduction and minimise the projection of fronto‐central negativity to posterior centro‐parietal channels (Kelly and O'Connell 2013; Twomey et al. 2015).

### 2.5 Pupillometry Acquisition and Pre‐Processing

An EyeLink 1000 eye‐tracking system (SR Research Ltd., Canada), with a desktop mount and infrared camera and illuminator, was used for real‐time monocular tracking at a sampling rate of 1000 Hz. The pupil size of the left eye was continuously recorded over the duration of each experimental block. A chin rest was used to aid head stability and minimise extraneous movements or off‐screen fixations. The eye‐tracker was calibrated and validated before each block using a 9‐point fixation sequence.

Pupillometric data were extracted and pre‐processed using MATLAB (version 9.22017a) software. First, the ‘.edf’ eye‐tracker files were converted to ‘.mat’ format, and the PD time series, sampling frequency, start times and blink indices were extracted. Stimulus event markers (i.e. target onset and probe presentation) were extracted from the behavioural files and converted to timestamps, as these were not present in the eye‐tracker output due to a technical error. Raw pupil data are commonly subject to artefacts or gaps from blinks or off‐screen fixations (Alnaes et al. 2014; Sirois and Brisson 2014). As such, a custom algorithm using MATLAB was employed to identify blinks (e.g. half‐blinks and pupil occlusion from eyelids or eyelashes) to add to the blink indices identified by the manufacturers' inline algorithm (see also van den Brink et al. 2016). Blink indices (extracted from .edf files) were expanded by 50 ms on either side to supplement the custom identification algorithm. Blinks and other artefacts, once marked, were removed and interpolated with a second‐degree polynomial curve‐preserving function. Visual inspection was also performed on all pupil data to verify the resulting pupil time series. Sessions that were deemed unfit for use (e.g. EyeLink failed recording and artefacts produced by correction of anomalies such as prolonged eye‐closing or sudden sustained changes in amplitude) were marked for rejection and were not used in further analyses (Section 2.1).

Pupil signals were low‐pass filtered at 6 Hz (Butterworth filter, 40th order, double‐filtered/zero‐phase shift) to remove higher frequency jitter and noise, including the steady‐state flicker frequency. Further, the pupil time series were *z*‐score normalised (within‐individual) before analysis. All single trials containing greater than 30% interpolated data points were rejected for both the target‐ and probe‐aligned analyses. As with the electrophysiological analyses, ‘Unintentional’ and ‘Intentional’ mind‐wandering responses were aggregated to form a total ‘Mind‐Wandering’ variable that was compared against ‘Focus’. Participants with fewer than 10 trials in the mind‐wandering condition remaining after pre‐processing procedures were further excluded from the analysis [younger adults, *n* = 1; older adults, *n* = 7].

For *target*‐*aligned analyses*, the pre‐target and post‐target epochs were extracted from −2000 ms to 0 ms and 0–4000 ms, respectively, and were baseline‐corrected with mean amplitudes calculated over the −200 ms to 0 ms pre‐target window. Moreover, *probe*‐*aligned* epochs were extracted from −2000 ms to 0 ms, time‐locked to probe presentation, and were baseline corrected with mean amplitudes calculated from the −2000 ms to −1800 ms pre‐probe interval. Pupil size was baseline corrected on a trial‐by‐trial basis, and these baseline corrected values were analysed rather than absolute measurements to account for possible inter‐individual differences in baseline pupil size and to attenuate habituation or the relative decline of pupil size over time (Sirois and Brisson 2014).

### 2.6 Signal Analysis

ERP componentry and oscillatory measures of EEG activity, and pupil dilation measures were investigated relative to target and probe stimuli onsets. Grand‐averaged waveforms were generated, and latency measurement windows were determined through visual inspection of the temporal and spatial extents of the components, and amplitude measures were isolated in accordance with previous research, the methods of which are described in the forthcoming paragraphs (see Alnaes et al. 2014; Dockree et al. 2017; Loughnane et al. 2019; McGovern et al. 2018; O'Connell et al. 2012; Sirois and Brisson 2014; Twomey et al. 2015). SSVEP, Alpha and PD were examined with respect to both target and probe stimuli, while the CPP and Mu/Beta signals traced decision‐related activity relative to target onset only. Data extraction and analysis procedures for each signal are henceforth discussed.

#### 2.6.1 Alpha: Attentional Engagement

##### 2.6.1.1 Target Analysis

EEG Alpha (8–14 Hz) was examined before target onset as an oscillatory measure of endogenous attentional engagement. Alpha amplitudes were extracted from a cluster of parietal and occipital channels (B6 and B7 for younger adults; A16 and B7 for older adults) as determined by the maximal amplitude activity of mean alpha power evident from the separate group scalp topographies [window: −2000 ms to 0 ms, relative to target onset]. For each participant, the mean pre‐target alpha amplitude was calculated over 20 cycles of the SSVEP (800 ms). The between‐trial variability of pre‐target alpha for each group was indexed by the CoV, quantified as the standard deviation of alpha amplitude divided by mean alpha activity. Alpha CoV was extracted for each participant as the primary alpha measure.

##### 2.6.1.2 Probe Analysis

Attentional modulation antecedent to probe onset was measured across the whole epoch by posterior alpha band activity (8–14 Hz) at channels A9, A10, B6 and B7 (younger adults) and B7 and B8 (older adults), identified from the grand‐averaged scalp topographies of mean alpha activity [window: −1800 ms to 0 ms, relative to probe presentation]. The mean alpha amplitudes for each participant were calculated using a static Fast Fourier Transformation (FFT) over 20 cycles of the SSVEP (800 ms) relative to each probe response. The CoV of alpha power was then calculated for each probe condition, namely pre‐focus and pre‐mind‐wandering (combined alpha amplitudes for the unintentional and intentional responses) and extracted as the primary alpha measure.

#### 2.6.2 PD: Attentional Engagement

##### 2.6.2.1 Target Analysis

Normalised PD was measured pre‐ and post‐target onset as a proxy psychophysiological marker of attentional engagement and LC‐NA activity (Aston‐Jones and Cohen 2005; Bang et al. 2023; DiNuzzo et al. 2019; Elman et al. 2017; Joshi et al. 2016; Meissner et al. 2024; Murphy et al. 2014; van den Brink et al. 2016). For the *pre*‐*target* normalised PD analyses, mean amplitudes were calculated across a temporal window of −2000 ms preceding target evidence onset for each participant. Further, the trajectory of the pre‐target normalised pupil changes was quantified by the pre‐trial slopes. Slopes were approximated with linear least squares fit on the individual epoched signals from −2000 ms to 0 ms, and the mean slopes were calculated across all trials for each participant. To examine *post*‐*target* normalised PD, both mean and peak amplitudes were extracted for each group from a window of 0 ms to 4000 ms, with respect to target onset, to gauge phasic pupil response to the target.

##### 2.6.2.2 Probe Analysis

For *pre*‐*prob*e normalised PD, mean amplitudes were extracted from a window of −1800 ms to −800 ms before focus and mind‐wandering conditions to avoid the checkerboard offset occurring at −500 ms.

#### 2.6.3 CPP: Decision Formation

##### 2.6.3.1 Target Analysis

The domain‐general CPP ERP component was recorded relative to target onset to trace the time course of sensory evidence accumulation and perceptual decision formation over target evolution, independent of preparatory motor dynamics (Kelly and O'Connell 2015; O'Connell et al. 2012). ERPs were generated by averaging single‐trial epochs for each participant that were combined to form grand‐averaged target‐locked CPP waveforms per group and low‐pass filtered using a fourth‐order digital Butterworth filter at 8 Hz for display only. The CPP was derived from a single scalp electrode site (channel A4) for both groups, identified from the grand‐averaged response‐locked scalp topographies as the region of maximal positive component activity [window: −150 ms to −50 ms relative to response] based on visual inspection. Two younger and two older adult participants displayed negative‐going CPPs with the selected electrode site; however, given that there was the same number of participants affected across groups, these data were included in the grand‐averaged data.

Amplitude measures of the ERP component were extracted from a broad latency window of 500–1750 ms relative to target onset; these measures included the peak magnitude (maximum positive voltage) and peak latency (timing of maximum positive voltage). The onset latency (start time) of the CPP was measured using a running point‐by‐point one‐tailed *t*‐test approach across time, looking at divergence from zero in a positive direction, examined separately for younger and older groups. For each group, the CPP onset latency was identified as the initial time‐point when the signal amplitude significantly diverged from zero (*p* < 0.05) and showed continuity of statistical significance above zero for at least the preceding 50 ms (see Loughnane et al. 2016, 2019). The build‐up rate of the CPP was quantified as the slope of a straight line fitted to the unfiltered stimulus‐aligned ERP waveforms over a window of 250–750 ms post‐target evidence onset. The temporal extent of the CPP was measured in line with previous research showing that stimulus changes require several hundred milliseconds before impacting the CPP build‐up rate (Kelly and O'Connell 2013, 2015; Loughnane et al. 2016; O'Connell et al. 2012).

#### 2.6.4 SSVEP: Sensory Evidence Encoding

##### 2.6.4.1 Target Analysis

The occipital SSVEP, driven by the intensity of the on–off stimulus flicker at a constant and rapid rate of 25 Hz, provided a continuous oscillatory measure that tracked basic visual stimulus processing and sensory evidence encoding. The SSVEP for channel selection was computed using a static FFT over a 20‐cycle window (800 ms) of the stimulus flicker frequency to reduce contamination by spectral leakage. Signal‐to‐noise (SNR) grand‐averaged scalp topographies [window: −100 ms to 0 ms, relative to target onset] were generated by dividing the power of the stimulus flicker frequency by the two adjacent frequencies in the frequency scale (i.e. 27.5 and 22.5 Hz) to enhance the specificity of the topographies. Guided by the regions of maximal SSVEP activity on the SNR topographies, amplitudes were averaged across three (A17, A21, A30) or two (A21, A22) channels, for younger and older groups respectively, centred on standard site POz for both groups.

The temporal evolution of the SSVEP (25 Hz) across the target‐locked epoch was calculated using the standard short‐time Fourier transform (STFT) procedure with a sliding boxcar window length of 400 ms for capturing an integer number of 10 cycles of the SSVEP frequency with a 20 ms step size. SSVEP measurements for each participant were normalised by dividing by the mean activity in the 250 ms pre‐target window. Normalised SSVEP target‐locked mean amplitudes were extracted from a window of 500–1600 ms relative to target onset to track sensory encoding until the point of peak physical evidence at 1600 ms. The build‐up rate was calculated as the slope of a straight line fitted to the unfiltered ERP waveforms over a window of 350–850 ms.

#### 2.6.5 SSVEP: Perceptual Decoupling

Traditionally, research has focused on the perceptual processing of the target trial measured, for example, by amplitude attenuation of the P300 component during mind wandering, which reflects a transient epoch of processing in time (see Smallwood and Schooler 2015). Here, we utilise the unfolding steady state 25 Hz signal as a continuous measure of perceptual processing prior to probes. This offers two potentially useful measures: (1) the temporal evolution of amplitude during mind wandering, which may indicate reduced processing of external stimuli indicative of perceptual decoupling and (2) amplitude variability, which captures the instability of perceptual processing during the pre‐probe state.

##### 2.6.5.1 Probe Analysis

The static FFT was measured within an 800 ms pre‐probe window for channel selection, starting at approximately −1600 ms and ending at −800 ms for each probe response, avoiding the checkerboard offset occurring at −500 ms. Guided by the grand‐averaged SNR scalp topographies [window: −1800 ms to −1500 ms, relative to probe onset], SSVEP amplitudes were averaged across three (A17, A21, A30) or two (A21, A22) channels, for younger and older groups respectively, centred on standard site POz for both groups.

The temporal evolution of the SSVEP (25 Hz) across the probe‐locked epoch was calculated using the STFT procedure with a boxcar window length of 400 ms capturing an integer number of 10 cycles of the SSVEP frequency with a 26 ms step size. The probe SSVEP measurements were not subjected to normalisation or further baseline‐correction; hence, between‐group analyses of pre‐probe SSVEP were not performed. Grand‐average SSVEP waveforms were generated for the different probe responses, namely, focus and mind‐wandering. Amplitude measures were extracted from a window of −1800 ms to −800 ms before focus and mind‐wandering trials and included the mean amplitude and amplitude variability (CoV, calculated as the standard deviation of SSVEP amplitudes divided by the mean activity). The build‐up rates of the SSVEP (slope) before each probe response were also calculated over approximately −1800 ms to −800 ms, relative to probe presentation.

#### 2.6.6 Mu/Beta: Motor Preparation

##### 2.6.6.1 Target Analysis

Effector‐selective motor preparation was indexed by oscillatory power in the mu/beta bands (8–30 Hz, excluding the 25 Hz stimulus flicker frequency) over motor regions in the left hemisphere (contralateral to the responding right hand). Based on the stimulus‐locked grand‐averaged topographies [window: −100 ms to 100 ms, relative to mean target response], LHB was averaged over three channels for older adults (D18–D20) and measured from one channel for younger adults (D19), centred for both groups on the standard left hemisphere motor site C3.

The time course of LHB power was measured using the standard STFT with a sliding boxcar window size fitting 10 cycles of the SSVEP frequency and a 20 ms step size. LHB amplitudes were normalised relative to the 250 ms pre‐stimulus window for each participant. Target‐locked normalised LHB mean amplitudes were examined within the window of 500–1250 ms relative to target onset, and the slope was measured over a target‐aligned window of 350 and 850 ms. Additionally, group differences in normalised LHB mean amplitudes and slopes were investigated within the window of 1000–1350 ms relative to target onset.

### 2.7 Statistical Analysis

Data normality was assessed using the Kolmogorov–Smirnov test. Extreme outliers, defined as values extending greater than three times the interquartile range (IQR) within a particular outcome measure per group, were precluded from further analysis. Outliers comprised fewer than 1.5% of all data points for both younger and older groups. Group comparisons on the spectral and time‐based EEG and pupillometric measures were assessed by a series of two‐tailed independent *t*‐tests. Where Levene's test for the equality of variances was violated, a Mann–Whitney *U* test was performed and associated tests with corrected degrees of freedom as well as median and IQR values were reported. To compare signal characteristics before different probe states, paired samples *t*‐tests were conducted separately for each group.

An exploratory 2 × 2 mixed repeated measures analysis of variance (ANOVA) was conducted to investigate the interaction between a between‐subjects factor of ‘Age Group’ and a within‐subjects factor of ‘Probe Response’ on SSVEP CoV amplitudes. Where the ANOVA revealed a non‐significant interaction, the main effects for the between‐ and within‐subjects factors were interpreted. Significant main effects were followed up with post hoc pairwise comparisons (paired samples *t*‐tests) to locate the source of the differences; Bonferroni‐corrected *p*‐values were reported to correct for multiple comparisons.

Exploratory two‐tailed partial Pearson's *r* correlations, controlling for age group, examined possible associations between neurophysiological and behavioural outcomes. A *p*‐value < 0.05 determined statistical significance. For each group comparison, Cohen's *d* effect size was calculated and interpreted as representing small (*d* = 0.2), medium (*d* = 0.5) or large (*d* = 0.8) effects (Cohen 1988).

Power analyses for the independent *t*‐tests revealed that our sample sizes of 25 younger and 30 older adults for the electrophysiological analyses and 33 younger and 31 older adults for the target pupil analyses were sufficient to detect large (*d* = 0.8) effects with greater than 0.83 and 0.88 probabilities, respectively. Calculations for the exploratory Pearson's *r* correlations, with one variable controlled (age group), an alpha cut‐off of 0.05, and an approximate sample size of 50 participants, provided 0.83 and 0.99 power values to detect medium (*r*_partial_ = ±0.4) and large (*r*_partial_ = ±0.6) effects, respectively. Although these calculations were performed after data collection, the effect sizes used were independent of the dataset and, therefore, not subject to the same biases as ‘post hoc’ power calculations computed with achieved effect sizes.

Bayesian analyses were also applied to complement the frequentist analyses to determine the presence or absence of a between‐groups effect and to support, particularly, the interpretation of non‐significant results. Bayes factor (BF), with a default scaling parameter of 0.707, yielded a relative measurement evaluating if the strength of the evidence favoured the predictive ability of the null over the alternative hypothesis (Dienes 2014). The evidence in favour of the null was interpreted as weak or inconclusive (BF_10_ = 0.33–3), moderate (BF_10_ = 3–10) or strong (BF_10_ > 10). A BF_10_ of less than one‐third provided evidentiary support for the null hypothesis; namely, that no group effect was present. SPSS Version 24 (IBM; Chicago, IL) and JASP software (JASP Team 2019) were used to conduct the frequentist and Bayesian analyses. Bar chart figures were generated in Prism 8 (GraphPad).

## 3 Results

### 3.1 Behavioural Performance

Behavioural performance indices and ES probe descriptive statistics for the total sample of 34 older and 34 younger adults (namely, those participants with data included in at least one of the neurophysiological analyses) were previously published (see Moran et al. 2021). No statistically significant between‐group differences were observed in mean RT, *t*(61.79) = −0.56, *p* = 0.580, 95% CI [−0.11, 0.06], *d* = 0.12, two‐tailed independent *t*‐test, BF_10_ = 0.28, Hit Rate, which was near ceiling for both groups, *U* = 432.50, *Z* = −1.07, *p* = 0.285, *d* = 0.27, Mann–Whitney *U* test, BF_10_ = 0.37 or the number of False Alarms, *U* = 383.50, *Z* = −1.55, *p* = 0.122, *d* = 0.39, Mann–Whitney *U* test, BF_10_ = 0.67. The groups did significantly differ, however, with respect to RT CoV; specifically, older adults responded to targets less variably than their younger counterparts, *t*(64) = 2.64, *p* = 0.011, 95% CI [0.72, 5.24], *d* = 0.65, two‐tailed independent *t*‐test, BF_10_ = 4.45.

With respect to the subjective data, older adults (*M* = 4.28, SD = 3.08) were less inclined to report total mind‐wandering incidences than younger adults (*M* = 7.13, SD = 2.91), *t*(65) = 3.88, *p* < 0.0005, 95% CI [1.38, 4.30], *d* = 0.95, two‐tailed independent *t*‐test, BF_10_ = 102.14. Older adults reported mind‐wandering in response to 26.78% of the probes, whereas younger adults reported mind‐wandering for 44.53% of the probes, on average. Partial Pearson correlations, presented in Moran et al. (2021), demonstrated that after controlling for Age Group and IQ (NART Errors), there was a statistically significant negative association between mind‐wandering and hit rate, *r*_partial_(56) = −0.41, *p* = 0.001, and a positive relationship between mind‐wandering and the number of false alarms, *r*_partial_(56) = 0.28, *p* = 0.034. Taken together, these results suggest that older adults exploited greater focus on the task, incurring a relative behavioural advantage via more stable performance (see also McGovern et al. 2018).

### 3.2 Target‐Aligned Neurophysiological Measures of Attentional Fluctuations

Neurophysiological markers of fluctuating attentional engagement (pre‐target alpha and PD) were measured relative to target onset for both age groups. Descriptive statistics for the between‐group comparisons on the target‐aligned EEG and pupil measures are displayed in Table 1. The total number of valid trials included in the target‐aligned alpha analyses differed significantly between younger (*M* = 181.48; SD = 95.40) and older adult groups (*M* = 229.17; SD = 70.28), *t*(52) = −2.11, *p* = 0.040, 95% CI [−93.05, −2.33], two‐tailed independent *t*‐test. However, between‐group analyses with a subset of randomly selected participants (representing 70% of the cases, with matched trial numbers across groups) found similar results to those using the total sample; therefore, the latter analysis using the total sample is reported. Further, there were no significant between‐groups differences in the number of trials included in the target‐aligned PD analyses between younger (*M* = 340.27; SD = 60.04) and older adults (*M* = 344.19; SD = 52.16), *t*(62) = −0.28, *p* = 0.782, 95% CI [−32.10, 24.26], two‐tailed independent *t*‐test.

**TABLE 1:** Descriptive statistics for the spectral and time‐based features of the target‐aligned neurophysiological measures of attentional engagement for younger and older adults.

Variable	Young		Old	
	*n*	*M* (SD)	*n*	*M* (SD)
Attentional engagement				
Pre‐target alpha CoV***	25	0.32 (0.07)	29	0.23 (0.03)
Pre‐target PD mean Amp***	33	0.02 (0.03)	31	−0.02 (0.05)
Pre‐target PD slope***	33	−0.01 (0.01)	31	0.01 (0.01)
Post‐target PD mean Amp*	33	−0.04 (0.09)	31	0.01 (0.02)
Post‐target PD peak Amp**	33	0.12 (0.16)	30	0.26 (0.18)

*Note:* Target alpha amplitude measure [window: −2000 ms to 0 ms]; pre‐target PD amplitude and slope measures [window: −2000 ms to 0 ms]; post‐target PD amplitude measures [window: 0–4000 ms]. *p < 0.05; **p < 0.01; ***p < 0.001.

Abbreviations: Amp, amplitude; CoV, coefficient of variance; M, mean; n, number of observations; PD, pupil diameter; SD, standard deviation.

#### 3.2.1 Pre‐Target Alpha

**FIGURE 2:**
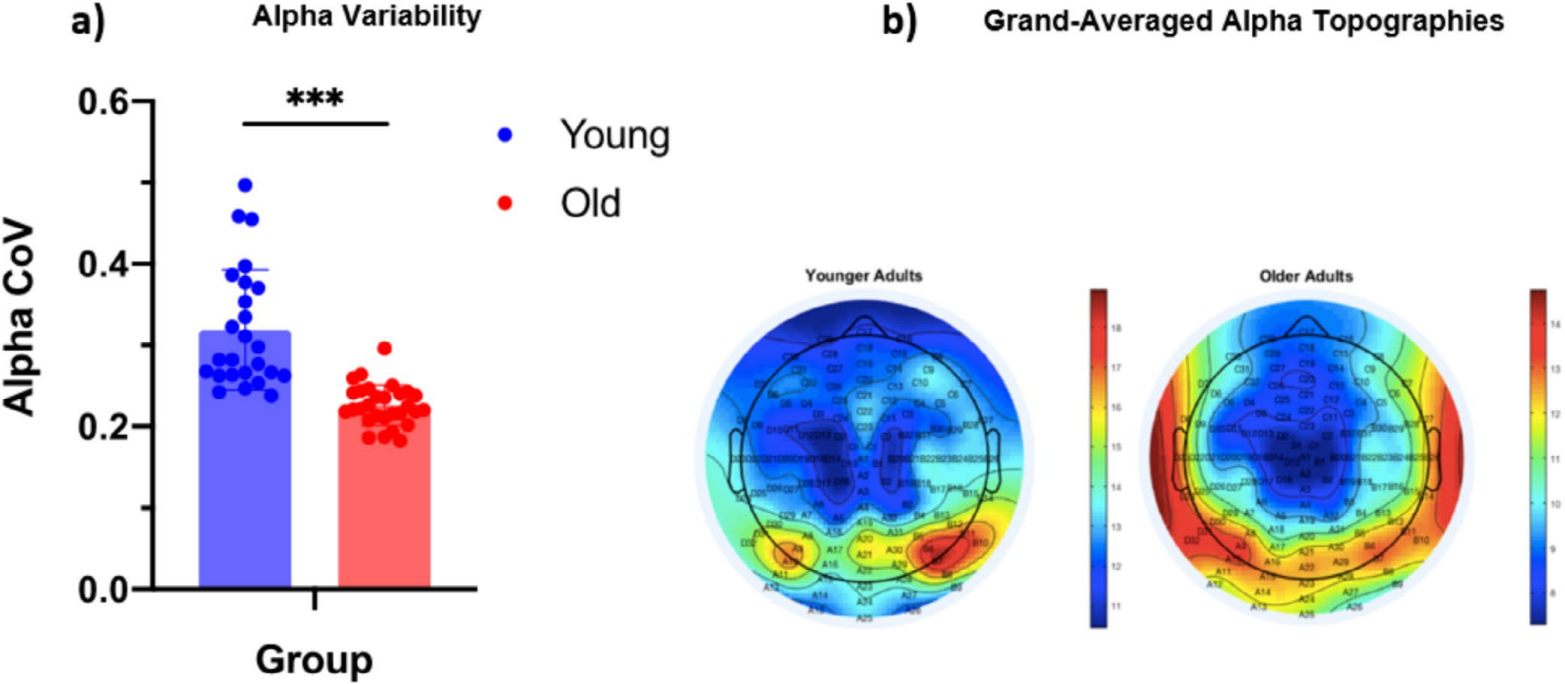
Bar chart comparing (a) pre‐target alpha variability and (b) alpha power scalp topographies for younger and older adult participants. (a) Bar chart comparing pre‐target alpha variability (coefficient of variance, CoV) between younger and older adults. Error bars represent the standard error of the mean (SEM). (b) Grand‐averaged topographies of mean occipital alpha power measured from −2000 ms to 0 ms preceding target onset for each group. There was no clear alpha peak for older adults, consistent with age‐associated reductions in alpha power and their steadier attentional engagement toward the task. ***p < 0.001.

Stimulus‐independent endogenous neural activity in spectral alpha power was explored before target onset during a window devoid of task‐evoked responses. A large and significant difference in pre‐target attentional modulation, assayed by alpha CoV, was observed between age groups, *t*(28.69) = 5.95, *p* < 0.0005, 95% CI [0.06, 0.12], *d* = 1.72, two‐tailed independent *t*‐test, BF_10_ = 168,138.38. Older adults exhibited less variable alpha‐band activity before target onset than their younger counterparts (Figure 2).

#### 3.2.2 Target‐Related Changes in PD

**FIGURE 3:**
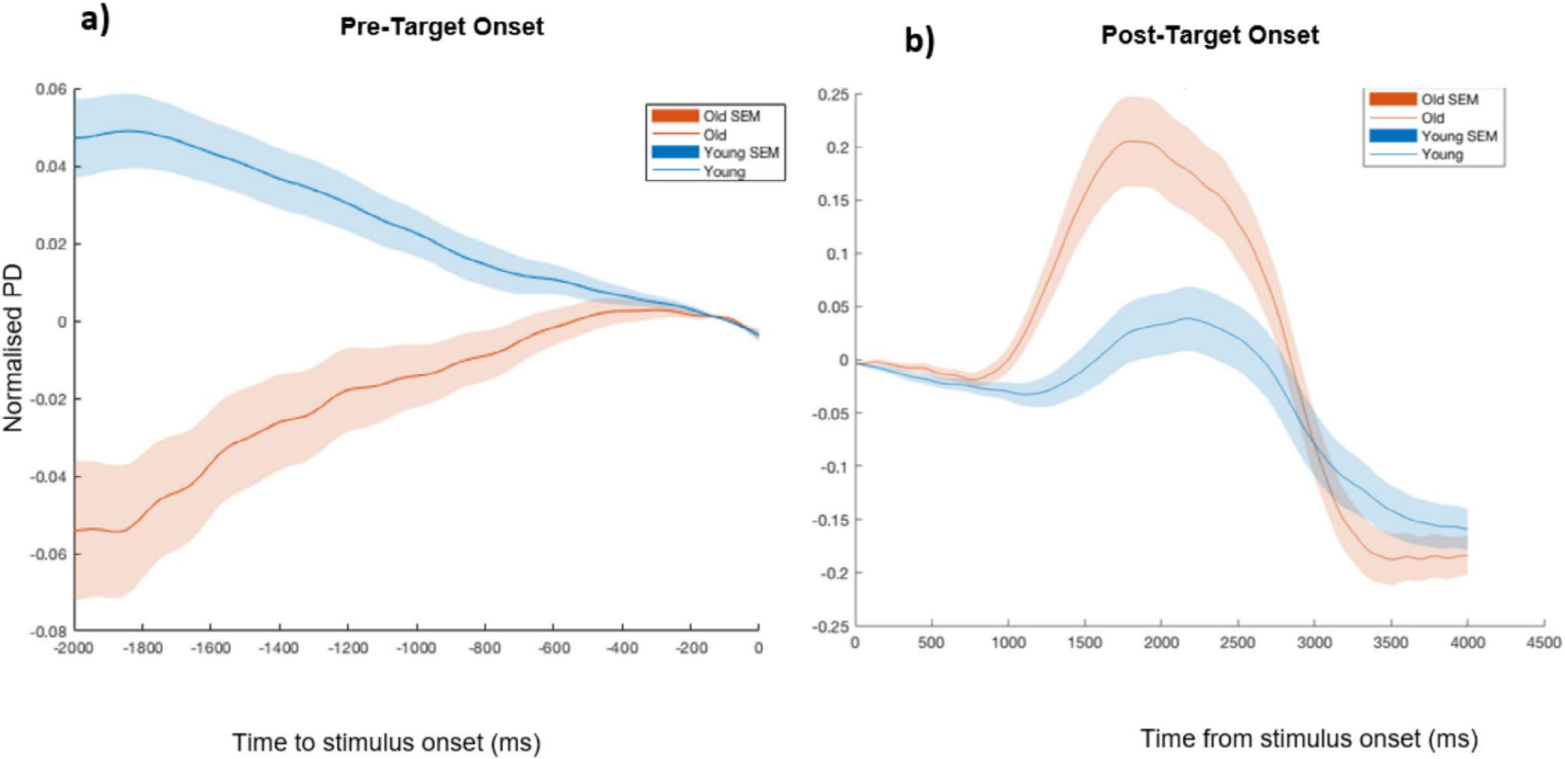
Target‐aligned grand‐averaged pupil diameter waveforms. (a) Pre‐ and (b) post‐target onset for younger and older adult participants. Grand‐averaged waveforms of normalised pupil diameter (PD) separated by group measured within a (a) *pre*‐*target* window of −2000 ms to 0 ms and (b) *post*‐*target* window of 0 ms to 4000 ms, where 0 ms represents target onset. Shaded areas indicate the SEM of data points.

An age‐related reduction in the mean amplitude of the normalised PD in the 2 s preceding target onset was observed, *t*(50.41) = 4.25, *p* < 0.0005, 95% CI [0.02, 0.06], *d* = 1.08, two‐tailed independent *t*‐test, BF_10_ = 351.62. The groups further differed with respect to the rate of pupil dilation changes, quantified by the mean slope, over the pre‐target period, *t*(62) = −5.71, *p* < 0.0005, 95% CI [−0.03, −0.01], *d* = 1.43, two‐tailed independent *t*‐test, BF_10_ = 36,145.73 (Figure 3a). Both groups display dynamic pupil changes in anticipation of the target, with younger adults demonstrating a more reactive response, shifting attentional engagement toward the task just in time before the onset of the target and older adults displaying a more preparatory and attentively engaged mode, as shown by the gradual rise in PD over the pre‐target interval.

The grand‐averaged normalised PD waveforms over the *post*‐*target* onset window tracked the full extent of the pupil response to targets, showing a reduction in arousal, marked by a reduction in normalised PD following peak evidence and response for both groups. Older adults demonstrated higher mean amplitudes, *t*(62) = −2.22, *p* = 0.030, 95% CI [−0.09, −0.01], *d* = 0.56, two‐tailed independent *t*‐test, BF_10_ = 1.98, and higher peak amplitudes, *t*(61) = −3.45, *p* = 0.001, 95% CI [−0.23, −0.06], *d* = 0.87, two‐tailed independent *t*‐test, BF_10_ = 30.00, relative to the shoulder of the response curve compared to younger adults (Figure 3b). Together, these target‐aligned pupil differences indicate that younger and older adults employ different task‐related strategies. Insofar as the PD response is an indicator of the LC‐NA system, older adults, compared to younger adults, show a more pronounced post‐target arousal response demonstrative of increased neural gain and task engagement.

### 3.3 Probe‐Aligned Neurophysiological Measures of Perceptual Decoupling

Since perceptual decoupling is proposed to reflect the capacity to redeploy attention away from sensory input to internal information (e.g. Smallwood 2013), the following were examined before different subjective attentional states within the pre‐probe interval: (1) the unfolding SSVEP, representing sensory input, (2) alpha, indexing sensory inhibition during attentional withdrawal from a primary task, and (3) PD, with its known relationship to exploitation/exploration dynamics linked to LC–NA function (Table 2).

**TABLE 2:** Within‐group descriptive statistics for the spectral and time‐based features of probe‐aligned neurophysiological measures of perceptual decoupling for younger and older adult participants.

Variable	Young				Old			
	Focus		Mind‐wandering		Focus		Mind‐wandering	
	*n*	*M* (SD)	*n*	*M* (SD)	*n*	*M* (SD)	*n*	*M* (SD)
SSVEP slope	22	0.00 (0.05)	22	0.00 (0.07)	18	−0.01 (0.05)	18	−0.00 (0.06)
SSVEP mean Amp	23	13.76 (4.21)**	23	14.26 (4.51)**	20	8.23 (3.62)	20	7.98 (3.22)
SSVEP Amp CoV	22	0.08 (0.01)*	22	0.10 (0.02)*	20	0.12 (0.02)	20	0.12 (0.03)
Alpha CoV	23	0.31 (0.08)*	23	0.34 (0.12)*	18	0.27 (0.05)	18	0.29 (0.05)
PD mean Amp	32	−0.03 (0.04)*	32	0.00 (0.06)*	24	−0.01 (0.07)	24	−0.02 (0.09)

*Note:* Pre‐probe SSVEP slope and amplitude measures [window: −1800 ms to −800 ms]; pre‐probe Alpha amplitude measure [window: −1800 ms to −800 ms]; pre‐probe PD amplitude measure [window: −1880 ms to −800 ms].

Abbreviations: Amp, amplitude; CoV, coefficient of variance; M, mean; *n*, number of observations; PD, pupil diameter; SD, standard deviation; SSVEP, steady‐state visually evoked potential.

* Significant within‐group differences at *p* < 0.05.

** Significant within‐group differences at *p* < 0.01.

After artefact rejection and pre‐processing, the number of trials contributing to the probe‐aligned SSVEP and alpha analyses was similarly matched across focused (*M* = 47.87; SD = 23.34) and mind‐wandering (*M* = 38.26; SD = 17.93) conditions for younger adults, *t*(22) = 1.48, *p* = 0.153, 95% CI [−3.85, 23.07], paired samples *t*‐test, BF_10_ *= 0*.*57*. The probe‐aligned SSVEP and alpha signal trial counts were, however, significantly different between focus (*M* = 59.35; SD = 24.58) and mind‐wandering (*M* = 32.60; SD = 20.65) conditions for the older adult group, *t*(19) = 3.18, *p* = 0.005, 95% CI [9.13, 44.37], paired samples *t*‐test, BF_10_ = 9.23. Trial counts were thus matched across conditions for younger adults but not for older adults, consistent with their decreased proclivity for mind‐wandering.

Additionally, there was no significant difference in the number of PD trials in the focus (*M* = 68.42; SD = 23.14) and mind‐wandering (*M* = 54.31; SD = 21.63) comparisons for younger adults, *t*(31) = 1.81, *p* = 0.079, 95% CI [−1.54, 26.29], paired samples *t*‐test, BF_10_ *= 0*.*81*. Although there was a significant difference in PD trial counts across focus (*M* = 84.77; SD = 27.06) and mind‐wandering (*M* = 39.00; SD = 20.03) conditions observed for older adults, *t*(23) = 5.41, *p* < 0.0005, 95% CI [27.59, 61.75], paired samples *t*‐test, BF_10_ = 1340.36. In these cases, we supplement the frequentist approach by specifically highlighting the Bayesian values for the older adult probe signal comparisons to determine whether the observed non‐significant findings are due to issues of statistical power (i.e. absence of evidence) or whether they support no effect (i.e. evidence of absence; Dienes 2014); these analyses are provided in the following sections.

#### 3.3.1 Pre‐Probe SSVEP

**FIGURE 4:**
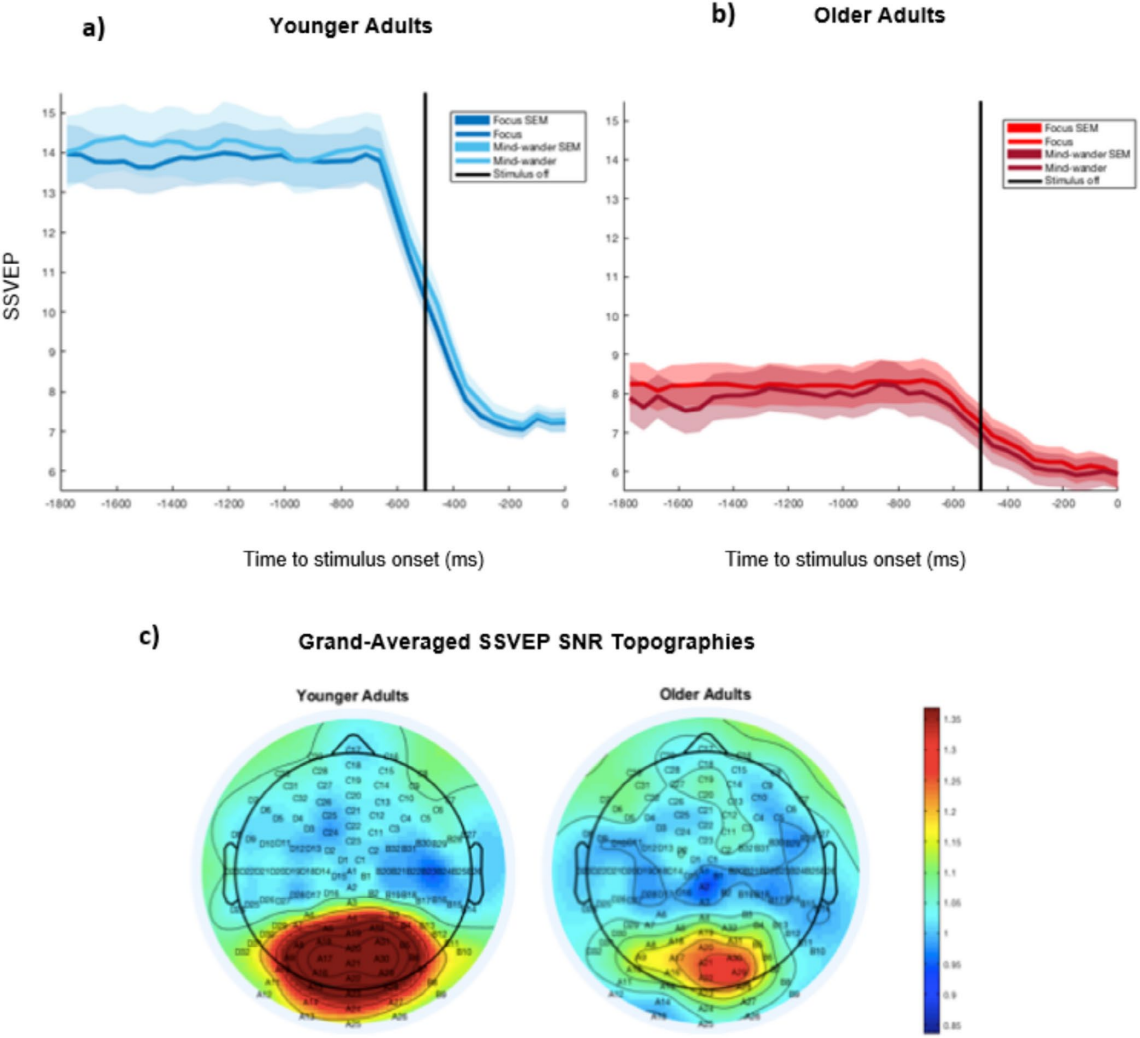
Probe‐aligned grand‐averaged signal waveforms showing sensory encoding (SSVEP) for (a) younger and (b) older adult participants and (c) grand‐averaged scalp topographies. Separate grand‐averaged waveforms of the steady‐state visually evoked potential (SSVEP) prior to focused and mind‐wandering states for (a) younger and (b) older adults, relative to probe onset at 0 ms. SSVEP amplitude and slope measures were extracted from a temporal window of −1800 ms to −800 ms, avoiding the checkerboard stimulus offset at −500 ms (denoted by the vertical black line). Shaded areas represent the SEM of data points. (c) Grand‐averaged scalp topographies of the signal‐to‐noise (SNR) SSVEP signal were measured from −1800 ms to −1500 ms preceding probe onset for younger and older adults and showed a positive component over occipital regions.

*Within*‐*group* comparisons of the SSVEP preceding focused and mind‐wandering states revealed no significant differences in the slope for either younger participants, *t*(21) = 0.03, *p* = 0.977, 95% CI [−0.04, 0.04], *d* = 0.01, paired samples *t*‐test, BF_10_ = 0.22, or older participants, *t*(17) = −0.38, *p* = 0.708, 95% CI [−0.05, 0.03], *d* = 0.09, paired samples *t*‐test, BF_10_ = 0.26. Bayesian analyses provided evidentiary support for the null hypothesis of no difference in SSVEP slope as a function of probe condition for either group. Contrary to expectation, higher mean SSVEP amplitudes were observed prior to mind‐wandering compared to focused states for younger adults, *t*(22) = −2.93, *p* = 0.008, 95% CI [−0.85, −0.15], *d* = 0.61, paired samples *t*‐test, BF_10_ = 6.12. No difference, however, was observed across conditions for older adults, *t*(19) = 1.55, *p* = 0.138, 95% CI [−0.09, 0.60], *d* = 0.35, paired samples *t*‐test, BF_10_ = 0.65; the BF value signals that this finding may be inconclusive (Figure 4).

An exploratory *within*‐*group* analysis revealed a significant difference in SSVEP amplitude CoV between focus and mind‐wandering for younger adults, *t*(21) = −2.45, *p* = 0.023, 95% CI [−0.02, −0.00], *d* = 0.52, paired samples *t*‐test, BF_10_ = 2.47, but not for older adults, *t*(18) = −1.02, *p* = 0.323, 95% CI [−0.02, 0.01], *d* = 0.23, paired samples *t*‐test, BF_10_ = 0.37 (representing a weak or inconclusive effect). Younger participants showed greater variability in the pre‐mind‐wandering sensory signal compared to pre‐focus, suggesting that they may pursue more intermittent sensory encoding when engaged in a relatively exploratory off‐task mode of thinking. Older adults, conversely, showed similar sensory evidence representation during focused and mind‐wandering states, providing some preliminary support for a more consistently exploitative task approach with age (Table 2).

A follow‐up exploratory 2 × 2 mixed repeated measures ANOVA revealed no significant interaction between Age Group and Probe Response Condition with respect to SSVEP CoV amplitudes, *F*_1,39_ = 0.87, *p* = 0.358, *ηp* ^*2*^ = 0.02, ANOVA. The main effect for Probe Response was significant, *F*_1,39_ = 5.82, *p* = 0.021, *ηp* ^*2*^ = 0.13, ANOVA, indicating that irrespective of group, SSVEP CoV differed between focused and mind‐wandering conditions. The Bonferroni corrected pairwise comparison showed greater variability pre‐mind‐wandering than pre‐focus, independent of Group, mean difference = 0.009, std. error = 0.004, *p = 0*.021, 95% CI (0.001, 0.016). Additionally, irrespective of probe response, younger and older adults significantly differed regarding SSVEP CoV, *F*_1,39_ = 33.85, *p* < 0.0005, *ηp* ^*2*^ = 0.47, ANOVA. Older adults displayed greater variability in the general pre‐probe sensory signal than younger adults, mean difference = 0.029, std. error = 0.005, *p* < 0.001, 95% CI (0.019, 0.039).

#### 3.3.2 Pre‐Probe Alpha

*Within*‐*group* paired samples *t*‐tests observed significantly greater alpha variability for mind‐wandering compared to focused conditions for younger adults, *t*(22) = −2.09, *p* = 0.049, 95% CI [−0.08, −0.00], *d* = 0.44, paired samples *t*‐test, BF_10_ = 1.35, but this difference was not similarly documented in older adults, *t*(17) = −1.58, *p* = 0.133, 95% CI [−0.05, 0.01], *d* = 0.37, paired samples *t*‐test, BF_10_ = 0.69 (weak or inconclusive effect).

**FIGURE 5:**
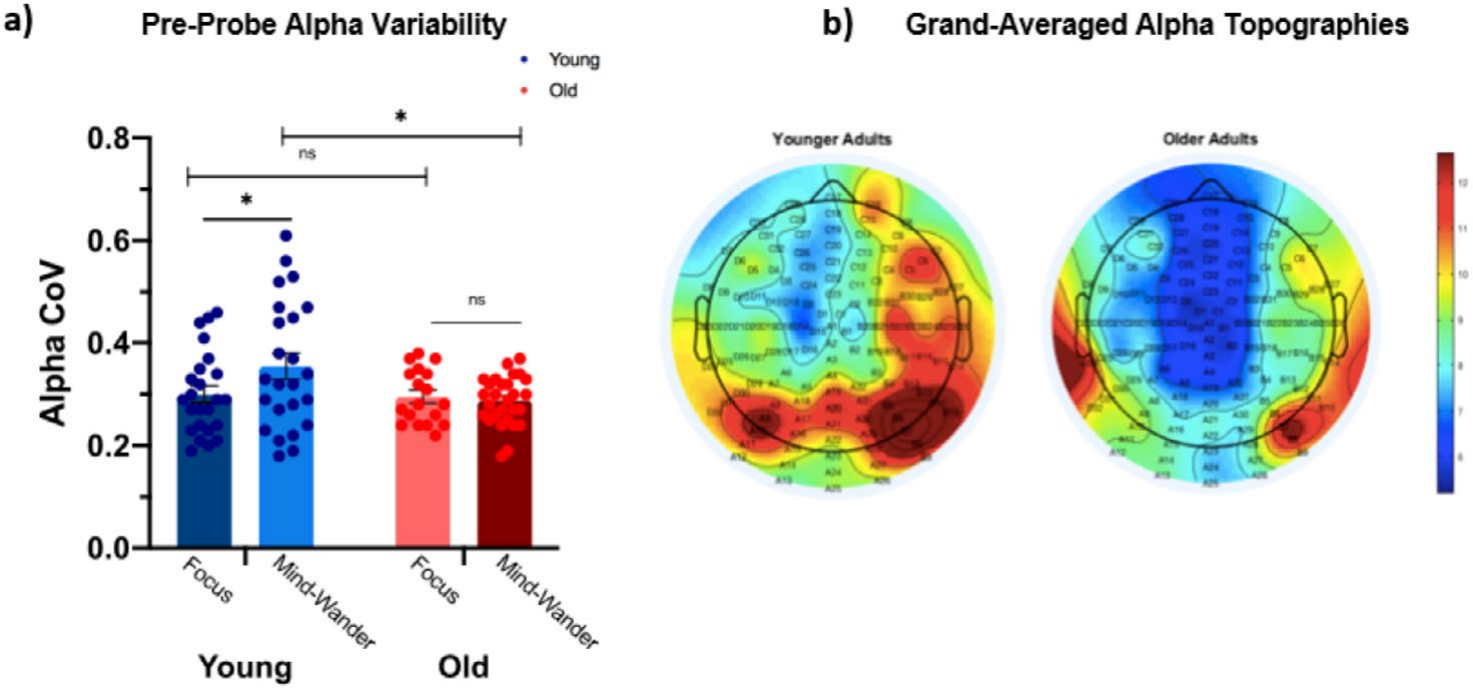
Bar chart comparing pre‐probe alpha variability (a) and alpha power scalp topographies (b) for younger and older adult participants. (a) Bar chart comparing probe‐aligned alpha variability (coefficient of variance, CoV) prior to focused and mind‐wandering states for younger and older adults. Younger adults exhibited greater alpha variability preceding mind‐wandering compared to Focus, but no difference between conditions was observed for older adults. Additionally, no between‐groups difference was observed in pre‐focus alpha CoV; however, older adults demonstrated less alpha variability prior to mind‐wandering than their younger counterparts. (b) Grand‐averaged topographies of the mean alpha signal [−1800 ms to 0 ms] relative to probe presentation for younger and older adults. ns: non‐significant; *p < 0.05.

An exploratory *between*‐*groups* analysis examined attentional modulation preceding probe presentation and demonstrated no significant difference between younger (*n* = 24, mean (*M*) = 0.30, SD = 0.08) and older adults (*n* = 28, *M* = 0.29, SD = 0.05) in Alpha CoV with respect to Focus, *t*(36.07) = 0.71, *p* = 0.485, 95% CI [−0.02, 0.05], *d* = 0.20, two‐tailed independent *t*‐test, BF_10_ = 0.35. There was, however, evidence to support a *between*‐*groups* difference in alpha CoV prior to Mind‐Wandering incidences, *t*(32.61) = 2.09, *p* = 0.045, 95% CI [0.00, 0.12], *d* = 0.59, two‐tailed independent *t*‐test, BF_10_ = 1.22. The reduced alpha variability in the older group (*n* = 18, *M* = 0.29, SD = 0.05), compared to younger adults (*n* = 24, *M* = 0.35, SD = 0.12), suggests a less marked shift away from an exploitative to an exploratory state with advancing age, even when mind‐wandering (Table 2, Figure 5).

#### 3.3.3 Pre‐Probe PD

A significant *within*‐*groups* difference in mean normalised PD before focus and mind‐wandering was observed for younger adults, *t*(31) = −2.49, *p* = 0.018, 95% CI [−0.06, −0.01], *d* = 0.44, paired samples *t*‐test, BF_10_ = 2.67, but not for older adults, *t*(23) = 0.49, *p* = 0.625, 95% CI [−0.03, 0.06], *d* = 0.10, paired samples *t*‐test, BF_10_ = 0.24. The BF for the older group effect provides evidence in favour of the null hypothesis, namely that there was no difference in PD between conditions for older adults.

**FIGURE 6:**
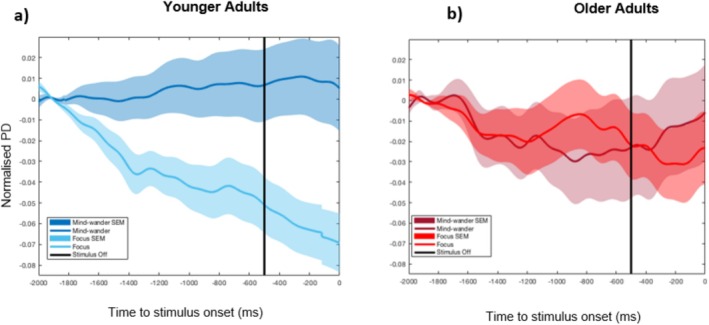
Probe‐aligned grand‐averaged signal waveforms showing attentional engagement (pupil diameter) prior to focus and mind‐wandering states for (a) younger and (b) older adults. Grand‐averaged waveforms of normalised and baseline‐corrected pupil diameter (PD) prior to focused and mind‐wandering states for (a) younger and (b) older adults relative to probe onset at 0 ms. Amplitude measures were extracted across a temporal window of −1800 ms to −800 ms to avoid the checkerboard stimulus offset at −500 ms, denoted by the vertical black line. The checkerboard offset was followed by a 500 ms blank screen before probe onset at 0 ms. Shaded areas represent the SEM of data points.

No significant *between*‐*groups* difference was observed in mean PD before Focus between younger (*n* = 33, *M* = −0.03, SD = 0.04) and older (*n* = 31, *M* = −0.01, SD = 0.07) adults, *t*(62) = −1.21, *p* = 0.233, 95% CI [−0.05, 0.01], *d* = 0.30, two‐tailed independent *t*‐test, BF_10_ = 0.47. Further, the data were inconclusive for a difference in pre‐Mind‐Wandering mean PD between younger (*n* = 32, *M* = 0.00, SD = 0.06) and older (*n* = 24, *M* = −0.02, SD = 0.09) adults, *t*(54) = 1.39, *p* = 0.170, 95% CI [−0.01, 0.06], *d* = 0.38, two‐tailed independent *t*‐test, BF_10_ = 0.61. Overall, there appeared to be a drop out of an explorative mode pre‐focus which was more accentuated in younger adults, as shown by a general rise in their PD before mind‐wandering in contrast with the general decrease in PD before Focus (Table 2, Figure 6).

### 3.4 Target‐Aligned Neurophysiological Measures of Perceptual Decision‐Making

**FIGURE 7:**
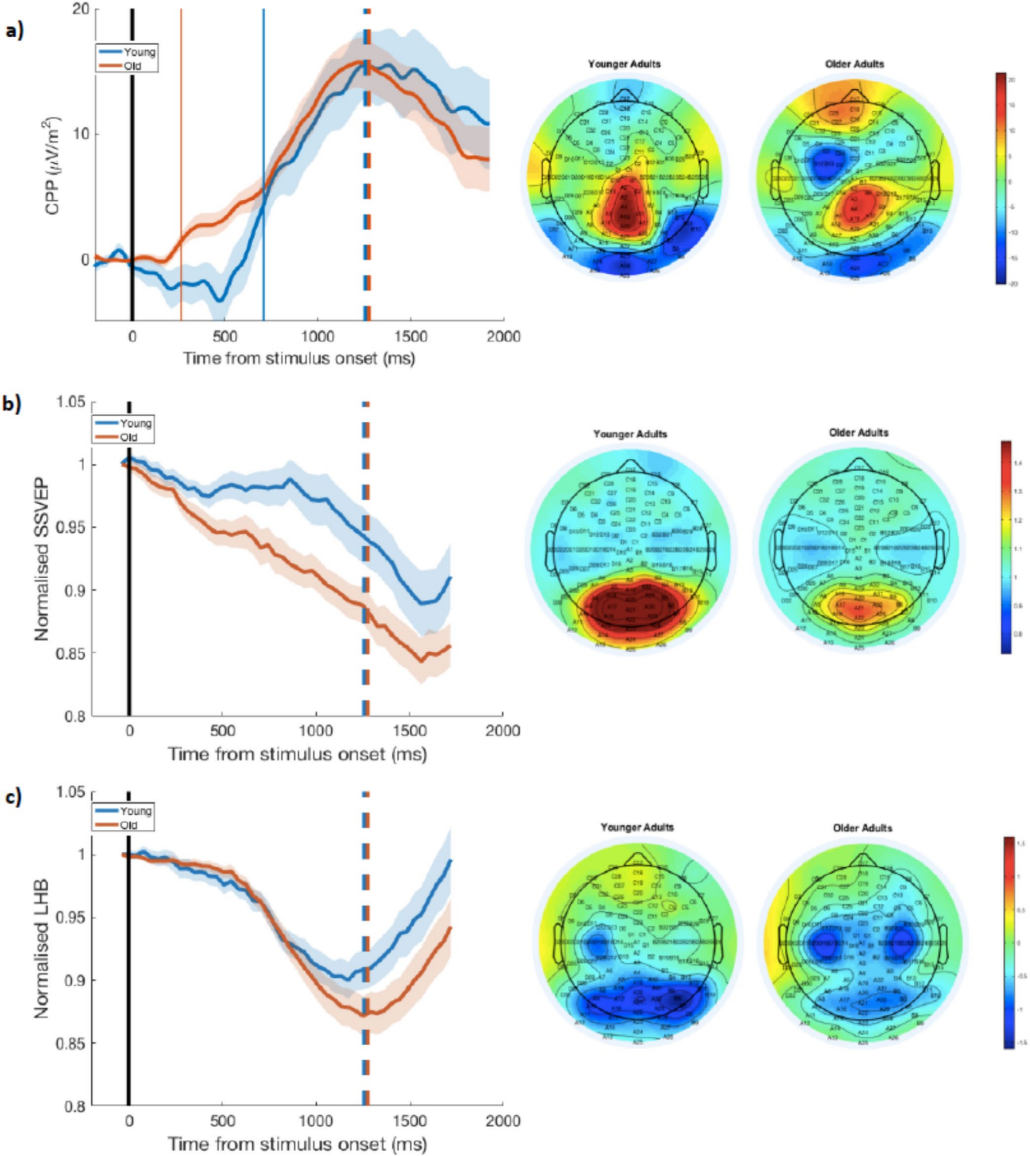
Target‐aligned grand‐averaged signal waveforms (left) and scalp topographies (right) showing (a) decision formation (CPP), (b) sensory encoding (SSVEP), and (c) motor preparation (mu/beta) for younger and older adult participants. Vertical lines at 0 ms denote the time of target stimulus onset. Dashed lines on the stimulus‐locked waveforms represent the mean reaction time for each group. Vertical coloured lines in panel (a) represent the CPP onset latencies for each group. Shaded areas indicate the SEM of data points. (a) Stimulus‐locked CPP waveforms separated by group plotted relative to target onset (left). CPP onset latencies for older and younger adults at 265.63 and 708.98 ms, respectively, are indicated by vertical coloured lines. Grand‐averaged topographies of the ERP signal −150 ms to −50 ms preceding response for each group show a positivity over centroparietal areas (right). (b) Stimulus‐locked normalised SSVEP waveforms separated by group plotted relative to target onset (left). Grand‐averaged topographies of the signal‐to‐noise SSVEP signal measured −100 ms to 0 ms preceding stimulus onset for each group show a large positive component over occipital regions (right). (c) Stimulus‐locked normalised LHB waveforms separated by group plotted relative to target onset (left). Grand‐averaged topographies of LHB signal measured −100 ms to 100 ms with respect to response for each group show maximal activity over premotor regions in the left hemisphere (right).

Neural indices of perceptual decision formation (CPP), sensory evidence encoding (SSVEP), and motor preparation (Mu/Beta) were examined at the time of target evidence onset and tracked over target evolution for younger and older adults. Descriptive statistics for the spectral and time‐based features of these decision‐related signal measures as a function of age group are provided in Table 3. Stimulus‐locked signal waveforms aligned to target onset and grand‐averaged scalp topographies for younger and older adults are displayed in Figure 7. The number of valid trials contributing to these decision‐related variables (i.e. for CPP, SSVEP and LHB) was not significantly different between younger (*M* = 147.84; SD = 94.66) and older (*M* = 186.00; SD = 92.95), *t*(53) = −1.50, *p* = 0.139, 95% CI [−89.07, 12.75], two‐tailed independent *t*‐test analyses.

**TABLE 3:** Descriptive statistics for the spectral and time‐based features of target‐aligned neurophysiological measures of perceptual decision‐making for younger and older adult participants.

Variable	Young		Old	
	*n*	*M* (SD)	*n*	*M* (SD)
Perceptual decision‐making				
CPP peak Amp	25	26.13 (15.93)	30	21.08 (11.26)
CPP peak latency, ms	25	1234.38 (223.31)	30	1318.16 (278.41)
CPP slope	25	0.01 (0.02)	30	0.01 (0.01)
SSVEP mean Amp*	25	0.96 (0.08)	30	0.90 (0.07)
SSVEP slope	23	−0.00002 (0.0001)	30	−0.00006 (0.0002)
LHB mean Amp	25	0.94 (0.05)	30	0.93 (0.05)
LHB slope	25	−0.0001 (0.0001)	30	−0.0001 (0.0001)
LHB mean Amp additional	25	0.91 (0.07)	30	0.88 (0.08)
LHB slope additional*	25	0.00002 (0.0002)	30	−0.0001 (0.0001)

*Note:* CPP amplitude measures [window: 500–1750 ms]; CPP slope [window: 250–750 ms]; target SSVEP amplitude measures [window: 500–1600 ms]; target SSVEP slope [window: 350–850 ms]; LHB amplitude measures [window: 800–1250 ms]; LHB slope [window: 350–850 ms]; Additional LHB amplitude and slope measures [window: 1000–1350 ms]. *p < 0.05.

Abbreviations: Amp, amplitude; CPP, centro‐parietal positivity; LHB, left hemisphere beta; M, mean; ms, milliseconds; *n*, number of observations; SD, standard deviation; SSVEP, steady‐state visually evoked potential.

#### 3.4.1 CPP During Target Evolution

Signal analyses demonstrated that the CPP at evidence onset did not vary with age (Figure 7a). There were no significant differences between younger and older adults in the peak amplitude of the CPP, *t*(53) = 1.37, *p* = 0.175, 95% CI [−2.32, 12.42], *d* = 0.37, two‐tailed independent *t*‐test, BF_10_ = 0.59; the peak amplitude latency, *t*(53) = −1.21, *p* = 0.230, 95% CI [−222.26, 54.68], *d* = 0.33, two‐tailed independent *t*‐test, BF_10_ = 0.50; or the rate of evidence accumulation (as measured through the build‐up rate of the CPP) *t*(32.76) = 1.23, *p* = 0.228, 95% CI [−0.00, 0.02], *d* = 0.35, two‐tailed independent *t*‐test, BF_10_ = 0.55. Overall, the Bayes Factors show that the data are inconclusive regarding possible statistical differences between groups in the integration of sensory evidence from target evolution until the decision threshold.

**FIGURE 8:**
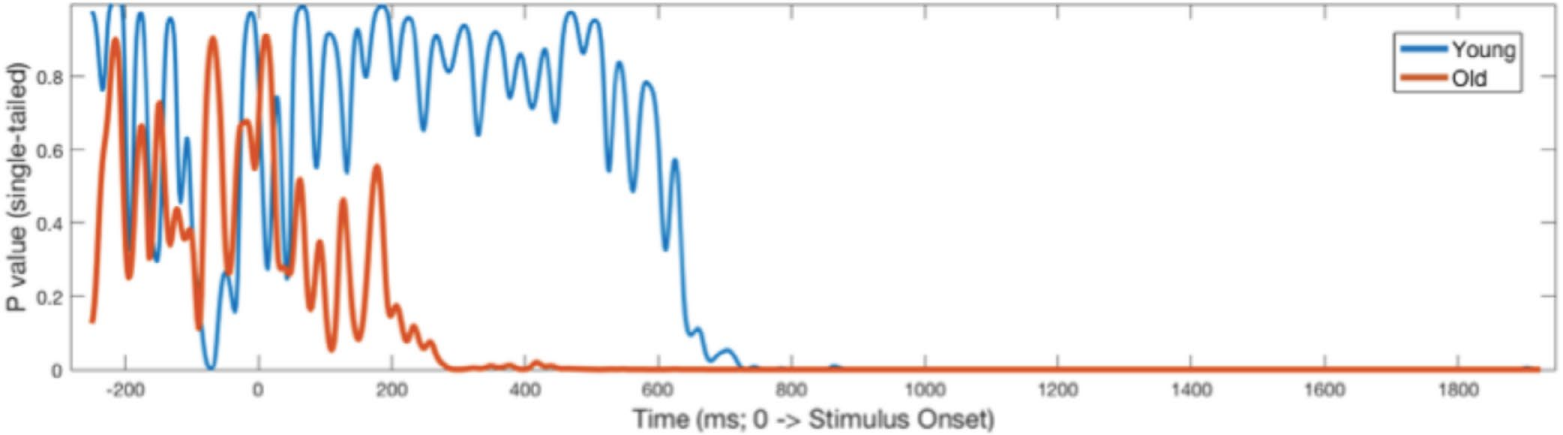
CPP onset: running point‐by‐point *t*‐tests against zero across the ERP waveforms. CPP Onset latencies were calculated for each group as the first time point the signal amplitude significantly exceeded zero in a positive direction (*p* < 0.05) and sustained this significant divergence over at least 50 ms. The CPP Onset latency for younger adults occurred at 708.98 ms, while older adults' onset latency occurred earlier at 265.63 ms. Figure 7a shows the grand‐averaged CPP waveforms from which these onset latencies were derived.

Differential group CPP onset latencies; namely, 708.98 and 265.53 ms for younger and older adults, respectively, revealed earlier initiation of sensory evidence accumulation for older adults. This pattern of earlier decision formation by older adults may reflect more exploitative, or conservative, perceptual evidence tracking with age and greater attentional readiness (Figures 7a and 8). Conversely, younger adults began integrating evidence of the stimulus change later, reacting when the contrast reduction became more salient.

#### 3.4.2 SSVEP During Target Evolution

The normalised SSVEP for both groups reliably traced the exogenously driven feature changes of the stimulus contrast. The grand‐averaged target‐aligned waveforms showed decreasing SSVEP magnitudes in line with the contrast reduction until full target evolution, that is, peak physical evidence at 1600 ms (Figure 7b). No group differences in the rate of sensory encoding, indexed by the normalised SSVEP slope, were observed, *t*(51) = 1.01, *p* = 0.317, 95% CI [−0.00004, 0.00012], *d* = 0.28, two‐tailed independent *t*‐test, BF_10_ = 0.42. A significant between‐groups difference, however, in the mean amplitude of the normalised SSVEP was revealed, *t*(53) = 2.54, *p* = 0.014, [0.01, 0.09], *d* = 0.69, two‐tailed independent *t*‐test, BF_10_ = 3.63. Here, the SSVEP traced the stimulus contrast reduction, so the finding of an age‐related reduction in mean SSVEP represents better sensory encoding with age. The more pronounced drop in mean SSVEP amplitude for older adults suggests that they more faithfully tracked the downward trajectory of the visual stimulus throughout the target period than their younger counterparts.

#### 3.4.3 LHB During Target Evolution

The effector‐selective LHB showed qualitatively similar age‐related patterns to the domain‐general CPP; namely, both groups demonstrated comparable decision‐related activity antecedent to full target evolution (Figure 7c). No significant differences in motor preparation were observed between younger and older adults either in the rate (slope), *t*(53) = 0.62, *p* = 0.539, 95% CI [−0.00004, 0.0001], *d* = 0.17, two‐tailed independent *t*‐test, BF_10_ = 0.32 or in the mean amplitude, *t*(53) = 0.51, *p* = 0.614, 95% CI [−0.02, 0.03], *d* = 0.14, two‐tailed independent *t*‐test, BF_10_ = 0.30, of normalised LHB over the target‐locked waveforms.

Additional analyses were performed to investigate possible differences in normalised LHB amplitude over a target‐locked time window around the time of mean RT (1000–1350 ms post‐target onset). No significant differences in LHB mean amplitude were observed between younger and older adults, *t*(53) = 1.31, *p* = 0.196, 95% CI [−0.01, 0.07], *d* = 0.36, two‐tailed independent *t*‐test, BF_10_ = 0.55. There was, however, a marginally significant difference in the LHB slopes between groups, *t*(53) = 2.01, *p* = 0.049, 95% CI [0.0000003, 0.0002], *d* = 0.55, two‐tailed independent *t*‐test, BF_10_ = 1.43; the BF, however, suggests that evidence for this effect is weak or inconclusive.

### 3.5 Exploratory Correlations Between Behavioural and Neurophysiological Measures

Exploratory partial correlations, controlling for Age Group, were performed to investigate the associations between behavioural performance indices and neurophysiological signals. Caution should be warranted in interpreting the results of these partial correlations as they are not corrected for multiple comparisons, but rather serve as exploratory analyses which may underpin future investigations. After controlling for Age Group, Hit Rate was negatively associated with pre‐target alpha CoV, *r*_partial_(48) = −0.40, *p* = 0.004, and with pre‐mind‐wandering mean PD, *r*_partial_(50) = −0.29, *p* = 0.036. Namely, better performance was related to steadier attentional engagement during the pre‐target and pre‐probe intervals, indexed by attenuated alpha variability and reduced pupil dilation. Additionally, hit rate was partially correlated with probe‐aligned PD difference scores (i.e. the difference in mean PD from mind‐wandering to Focused states), *r*_partial_(50) = −0.33, *p* = 0.016, suggesting that enhanced accuracy was associated with less shifting from exploitative to explorative modes.

Reciprocally, self‐reported Mind‐Wandering frequency was positively partially associated with pre‐target mean SSVEP, *r*_partial_(51) = 0.27, *p* = 0.049, pre‐mind‐wandering mean PD, *r*_partial_(53) = 0.28, *p* = 0.042, and with probe‐aligned PD difference scores, *r*_partial_(53) = 0.27, *p* = 0.047. That is, less efficient target stimulus encoding, elevated pupil size, and more marked pupil changes between subjective attentional states were individually associated with greater mind‐wandering propensity, independent of Age Group.

When the age group was controlled, larger RTs (slower performance) were positively associated with widened and delayed decision policy indexed by higher CPP peak amplitudes, *r*_partial_(52) = 0.32, *p* = 0.018, and delayed CPP peak amplitude latencies, *r*_partial_(52) = 0.41, *p* = 0.002. Moreover, the mean LHB slope extracted from the temporal window around the time of the mean RT (1000–1350 ms) was negatively associated with mean RT, *r*_partial_(52) = −0.43, *p* = 0.001. Further, RT CoV was negatively associated with CPP peak amplitude latency, *r*_partial_(51) = −0.32, *p* = 0.019, and with post‐target mean PD amplitude, *r*_partial_(59) = −0.28, *p* = 0.030. Fluctuating performance was therefore linked with a less robust response to target evolution, with earlier, and perhaps more impulsive, perceptual decision formation. Additionally, RT CoV was positively partially correlated with pre‐Focus SSVEP CoV, *r*_partial_(46) = 0.29, *p* = 0.043, and with probe‐aligned PD difference scores, *r*_partial_(51) = 0.28, *p* = 0.045. As such, performance variability was associated with more variability in the sensory signal before focused states, and with greater exploratory shifting during the pre‐probe interval. Finally, the number of false alarms was positively associated with the mean amplitude of normalised LHB, *r*_partial_(49) = 0.29, *p* = 0.041. False Alarms were related to reduced beta desynchronisation, possibly indicating greater uncertainty or a more liberal threshold for motor response preparation and execution based on less evidence. The behavioural outcomes were not further significantly correlated with any of the other neurophysiological signals.

## 4 Discussion

We advance four key findings that indicate an age‐related difference in oscillatory strategies, propounding a more exploitative task approach with advancing age.

1. Steadier attentional engagement preceding target onset was observed for older adults compared to their younger counterparts, as seen by attenuated variability in the attentional signal and a more robust phasic PD response to the target.
2. Younger, but not older, adults exhibited perceptual decoupling through more intermittent sensory encoding, indexed by greater variability in the sensory and attentional signals before mind‐wandering relative to focused states.
3. Older adults demonstrated earlier onset of evidence accumulation and better sensory representation of the target stimulus compared to younger adults. In further support of perceptual decoupling for younger adults, they showed delayed and reduced representation of the sensory evidence as it evolved over the target trial. This suggests that younger adults were less adept at tracking the sensory evidence at the critical point where the contrast changes because they were more generally disengaged.
4. Increased attentional variability and attenuated task‐related processing were associated with performance decrements and increased mind‐wandering, independent of age group, suggesting the implications of transitional shifts between exploitative and explorative states.

Considering the potential implications of oscillatory attentional engagement, the age‐related difference in mind‐wandering propensity, and group equivalence in task performance, we contend that younger and older adults employed different task‐related strategies. Younger adults flexibly alternated between competing goal‐directed and explorative strategies without incurring relative performance costs, despite their increased mind‐wandering propensity and more variable sensory evidence encoding and attentional engagement. Older adults marshalled their more limited cognitive resources toward the task and showed less bias for exploration, even during mind‐wandering. This was supported by their reduced mind‐wandering tendency, steadier attentional engagement pre‐target and pre‐Mind‐Wandering, and more conservative decision policy, compared to younger adults.

### 4.1 Steadier Attentional Engagement With Advanced Age

Prior to target onset, older adults demonstrated more stable attentional engagement, as shown by reduced posterior alpha band variability, coupled with attenuated mean normalised PD and gradually increasing pupil dilation in anticipation of the target. These antecedent attentional markers were examined over a temporal window absent of task‐dependent target changes, which enabled the investigation of intrinsic neural activity that was less impeded by stimulus‐evoked responses. Following target presentation, older adults exhibited higher mean and peak normalised PD amplitudes, suggesting that they tracked the full extent of target evolution with greater integrity. This is in line with Joshi et al. (2016) who showed a linear relationship between LC and pupil size, namely that greater PD is linked to more NA in the service of goals. Indeed, there is a growing literature to show that PD is a reliable proxy measure for LC activity across several task contexts (Bang et al. 2023; DiNuzzo et al. 2019; Elman et al. 2017; Meissner et al. 2024; Murphy et al. 2014). Conversely, younger adults showed a steep decrease in PD prior to target onset, suggesting that they dropped out of an exploratory mode prior to target onset with further attenuated pupil response to targets. These age‐related patterns are further substantiated by the behavioural finding of reduced RT CoV in older adults.

Together, these findings suggest a more restricted exploitative oscillation strategy with age, conferring a relative behavioural advantage for older adults through steadier performance and better attentional engagement. Indeed, a bias for explorative tendencies in younger adults has been previously reported (Mata et al. 2013). These findings also replicate an effect formerly observed by McGovern et al. (2018), in which less variable alpha activity and RT performance were documented in older adults compared to younger adults. This was demonstrated using a previous variant of the current task without the inclusion of probes, and as such, the age‐related discrepancy in attentional dynamics and strategic policies does not appear to be consequences of periodic task disruptions from ES procedures.

### 4.2 Younger Adults Show Perceptual Decoupling During Mind‐Wandering

Signal analysis within the pre‐probe interval provided support for perceptual decoupling (Smallwood and Schooler 2006, 2015) in younger, but not older, adults. Specifically, younger adults exhibited more intermittent sensory encoding (greater SSVEP CoV) in conjunction with more variable synchronisation of the attentional signal (alpha CoV) during self‐reported mind‐wandering states relative to focused states. Endogenous baseline PD was also analysed as a proxy psychophysiological measure of LC‐NA neuromodulatory activity, representing a potential mechanism through which brain states may flexibly shift between competing exploit/explore modes. Younger adults displayed higher mean PD amplitudes prior to mind‐wandering compared to Focus and appeared to drop out of an exploratory mode pre‐focus as evidenced by their more accentuated rise in PD prior to mind‐wandering and decrease in PD prior to focus.

Younger adults also displayed higher mean SSVEP prior to mind‐wandering relative to Focus. Given that previous research has shown reduced electrophysiological responses to external events in perceptual decoupling, the reverse effect observed was unexpected. However, we speculate that if the 25 Hz sensory signal was processed with greater neural efficiency during Focused states, then more desynchronised neural populations may have yielded a reduced SSVEP amplitude. Indeed, cognitive efficiency theories often predict that reduced amplitudes index better visual processing efficiency (Rypma et al. 2002; Wiegand et al. 2014). It follows that a loss of neural efficiency during mind‐wandering may have induced more synchronised neural activity, increasing the SSVEP amplitude. However, it is important to note that previous research into decoupling has focused on the perceptual processing of target stimuli, and in this regard, our target‐aligned results show a similar outcome via less target sensory evidence and more mind‐wandering in younger adults. Furthermore, it might be that when sensory evidence is stable (i.e. unchanging in contrast) as is the case with the pre‐probe states, an index of variability may better capture how efficiently the 25 Hz flicker is sampled. Indeed, the present CoV measure picked up greater variability prior to mind‐wandering than focused states for younger adults.

Prominent previous work has similarly shown elevated posterior alpha activity prior to lapses in attention (O'Connell et al. 2009) and during subjective mind‐wandering states (Compton et al. 2019), internally oriented attention (Braboszcz and Delorme 2011) or at rest (Laufs et al. 2003), suggesting the influence of top‐down processes on sensory inhibition (Klimesch 2012). This is further corroborated by studies reporting compromised sensorimotor processing of perceptual events via attenuated electrophysiological and pupillary responses to sensory input during or preceding mind‐wandering (Baird et al. 2014; Barron et al. 2011; Braboszcz and Delorme 2011; Kam et al. 2011; Macdonald et al. 2011; Smallwood et al. 2011; Walpola et al. 2020), as well as pupillary changes preceding explorative behaviours (Jepma and Nieuwenhuis 2011).

Contrary to expectations, however, there was no clear evidence of momentary reductions in the magnitude or rate of the sensory signal during mind‐wandering compared to focus for older adults. Older adults maintained similar levels of sensory representation and attentional engagement prior to both subjective focus and mind‐wandering states, supporting a more conservative strategic approach with age. Moreover, the age‐related reduction in pre‐mind‐wandering alpha CoV, which was not similarly evident pre‐focus, highlights a less pronounced shift away from the exploit to the explore state by older adults when mind‐wandering is reported. Given the temporally unpredictable nature of the target onset, older adults showed a tendency for more cautious, continual task monitoring to ensure adequate performance. However, given the low incidence of mind‐wandering for older adults, and as a consequence, low trial counts, these non‐significant results may have been subject to issues of statistical power.

### 4.3 Earlier Evidence Accumulation and Greater Sensory Encoding by Older Adults

Dissociable stimulus‐aligned neural indices of perceptual decision formation, sensory evidence encoding, and motor preparation demonstrated that both age groups reliably and similarly tracked the exogenous feature changes of the target contrast reductions as they evolved over time (see also McGovern et al. 2018; O'Connell et al. 2012). Although previous studies have demonstrated decreased P3 amplitudes and increased peak latencies (Fjell and Walhovd 2001; Rossini et al. 2007) and stronger mu/beta desynchronisation with age (Quandt et al. 2016; Sailer et al. 2000), we observed no marked group differences in these perceptual decision‐making components. In contrast to earlier studies, our task differs insofar as targets gradually evolve in time over 1.6 s, reducing rapid information processing demands upon older adults while increasing demands on the sustained attention system. We observed only a weak effect showing greater beta power desynchronization via a more pronounced slope for older adults around the time of mean RT, consistent with older adults maintaining an exploitative approach; this was not further supported by the between‐group analyses of mean amplitude measurements or the examination of LHB slopes during the broader target‐locked time window. However, our results are broadly consistent with McGovern et al. (2018) who used a variant of the present task and observed similar target processing across groups with the exception of an age‐related difference in the variability of the CPP build‐up rate.

In this study, older adults exhibited earlier CPP onset and more faithfully tracked the downward trajectory of the contrast reduction throughout the target period, as indexed by reduced mean target‐related SSVEP amplitude. These patterns of earlier initiation of evidence accumulation accompanied by enhanced sensory encoding of the target stimulus are consistent with older adults adopting a more cautious decision policy and engaging in more persistent perceptual monitoring to prevent missing targets. This is supported by drift diffusion models showing wider and more conservative decision boundaries with age (Ratcliff et al. 2006, 2010; Starns and Ratcliff 2010).

Conversely, the delayed onset in younger adults was indicative of more reactive temporal integration of sensory evidence, which was postponed until the evidence was more salient; this may have occasioned more opportunity for younger adults to engage in mind‐wandering without impacting overall performance. The delayed and reduced sensory evidence representation during the target trials provides additional support for perceptual decoupling in younger adults; namely, they integrated the target stimulus sensory evidence less efficiently due to their more frequent attentional disengagement.

### 4.4 Poorer Performance Accompanied by Attentional Fluctuations, Independent of Group

Our exploratory partial correlations demonstrated the disruptive influence of fluctuating attentional states on neurocognitive processing and behavioural performance, independent of Age Group. In line with perceptual decoupling, top‐down stimulus‐independent (oscillatory alpha activity) neural activity and bottom‐up task‐evoked (attenuated decision formation and variable sensory evidence representation) neural activity were associated with greater withdrawal from the primary task. Specifically, enhanced performance accuracy and reduced mind‐wandering were associated with steadier attentional engagement in the pre‐target and pre‐probe intervals, indexed by reduced pre‐target alpha variability, pre‐mind‐wandering pupil dilation, and less pronounced strategic shifting between probe states (indexed by PD difference scores).

Performance decrements were also associated with reduced efficiency of task‐related processing, controlling for age group. Specifically, delayed decision formation and reduced motor preparation were associated with slower responding and increased false alarms, respectively. Less performance variability (RT CoV) was linked with earlier decision formation and with greater post‐target pupil dilation in the target interval. Taken together, fluctuating performance was accompanied by a less robust response to target evolution and earlier, impulsive perceptual decision formation (see also Dockree et al. 2017; Kelly and O'Connell 2013; O'Connell et al. 2009). Additionally, greater performance variability was associated with probe‐aligned variables including more variable sensory evidence encoding prior to Focused states and with greater exploratory shifting during the pre‐probe interval. These findings suggest that oscillating attentional engagement and explorative states have implications for task‐related processing and performance.

### 4.5 Theoretical Implications

With respect to the present findings, we observed that fluctuating attentional states are accompanied by disrupted behavioural performance and reduced task‐related neural processing, independent of Age Group. In light of the potential consequences of attentional withdrawal from a task, our finding of group parity in overall task performance suggests that younger and older adults employed different task strategies to mitigate performance costs, in line with the exploitation/exploration framework (Sripada 2018). Indeed, we found younger adults displayed a greater tendency for exploration, flexibly shifting between goal‐directed and mind‐wandering processes without a corresponding decline in performance. Despite younger adults' increased propensity for mind‐wandering and evidence of more variable responding, sensory evidence encoding, and attentional engagement, they appeared to utilise their greater cognitive resources to balance these serial modes more optimally. Conversely, older adults compensated for their reduced cognitive capacity by dedicating their resources to the task in a more exploitative manner, prioritising task‐relevant information, and suspending mind‐wandering to mitigate potential performance costs. Mind‐wandering may confer benefits or consequences depending on how it is applied relative to the demands of the ongoing context. Affordance of an opportunity to mind‐wander when the context allows promotes meaning‐making, generative modes of creativity and problem‐solving, and underpins reflective recollection and anticipatory prospective modes of thinking. There is also evidence that mind‐wandering during the day might allow more efficient memory consolidation processes in a similar way to REM sleep at night (see Brokaw et al. 2016). However, if a reduction of mind‐wandering is conceived as an age‐related deficit in the flexibility of shifting between exploit and explore strategies, it may have negative implications, for example, as Sripada (2018) would suggest, namely via a less balanced oscillatory mode in daily life. It follows that if mind‐wandering generally reduces with age (and possibly, not only during strategically helpful situations) but due to general Default Mode Network (DMN) decline, then it could undermine the processes that give rise to self‐generated mental content. For example, some dementia patients show reduced mind‐wandering and increased stimulus‐bound thought accompanied by reduced structural and functional integrity of the DMN (O'Callaghan et al. 2019). However, if mind‐wandering is strategically limited by older adults to maintain performance, this may be seen as an adaptive quality of ageing.

### 4.6 Methodological Considerations

A key methodological advantage is that the target contrast changes unfolded smoothly and gradually, thereby eliminating sensory‐evoked deflections from the signal recordings, circumventing exogenous attention capture and placing greater dependence on endogenous attentional control. Our novel paradigm enabled isolation and investigation of the complex and dynamic properties of mind‐wandering.

Despite the advantage of ES probes, we are cognizant that these reports are not arrived at objectively but are formed from a first‐person viewpoint. Subjective reports may be influenced by researcher‐imposed or self‐interpreted definitions of mind‐wandering, probe‐framing and probe‐timing, and meta‐awareness capabilities, factors that are influenced by ageing (Jordao et al. 2019). Specific challenges have been raised in the literature regarding taking an introspective approach, including whether the probe gives veridical descriptions of the phenomenological states it purports to measure, and secondly, whether the act of receiving and responding to a probe interrupts performance. In response to the first point, intermittent probe sampling employed in the present study enabled qualitative categorisation of subjectively reported mental states at discrete moments in time to facilitate comparisons of behavioural patterns and neural activity between on‐ and off‐tasks. In Moran et al. 2021 we showed that the probes were meaningfully related to behavioural and neuropsychological concomitants (see also Cheyne et al. 2009; McVay and Kane 2009; Mooneyham and Schooler 2013) and we further demonstrated associations between self‐reported Mind‐Wandering frequency and neurophysiological signals in both the pre‐target and pre‐probe epochs (see also Frank et al. 2015; Golchert et al. 2017). Hence, there is little reason to suspect that the probes are not tapping into key brain processes even if there are certain features of the experiences not currently measured by the present approach.

Regarding the second point, Simola et al. (2023) showed that basic features of brain responses (parietal P3 amplitudes) to tasks were independent of the presence of ES probes, suggesting that introspection does not majorly disrupt neural processing. Additionally, our observed age‐related difference in RTV, suggestive of steadier attentional engagement by older adults, replicated an effect originally reported by McGovern et al. (2018) who utilised a variant of the current task without the inclusion of the ES probes. Specifically, McGovern et al. (2018) reported greater variability in CPP build‐up coupled with more variable posterior alpha band activity in younger adults compared to older adults, indicative of a higher degree of attentional fluctuation, supporting the age‐related RTV discrepancy. Given the links between RTV and variable EEG states in studies that did (the present study) and did not (McGovern et al. 2018) employ ES procedures, variability in task performance does not appear to be a consequence of task interruptions but rather, it appears to be driven by age‐related differences in oscillating attention. Taken together, these findings support the validity of ES as a tool for gauging phenomenologically different mental states without introspection unduly disrupting the flow and quality of the mental experience.

We contend, however, that there may be alternate relevant mental experiences (e.g. external distractions, mind‐blanking, ruminative thoughts, etc.) that were not captured by the probe response options employed in the present study that fall under the broad spectrum of attentional lapses and mind‐wandering. For example, Moran et al. (2021) found that greater mind‐wandering was correlated with more anxiety (HADS‐A) and greater ADHD‐like symptoms as measured by the CAARS. These more hyper‐aroused states are characteristic of scattered thoughts and labile emotions that may promote exploratory mental states. In future work, multi‐dimensional experience sampling (MDES) may be useful to examine other facets of internal mental experience. For example, Simola et al. (2023) demonstrated through the use of MDES that reductions in external task focus were related to social episodic thoughts. Indeed, this reflects the ongoing debate and lack of consensus surrounding mind‐wandering terminology (see Christoff et al. 2016, 2018; Seli, Kane, et al. 2018; Smallwood and Schooler 2015). The present contributions are contextualised by the specific non‐demanding, continuous sustained attention lab task paradigm. Mind‐wandering propensity should be further investigated across a range of task paradigms where task demands and cognitive load are manipulated to assess the degree to which an individual prioritises task‐related information versus self‐generated thought during differentially demanding tasks (Seli, Konishi, et al. 2018; Turnbull et al. 2019). Additionally, the present task presented little semantic variability, and therefore, the amount of subjective input prompting mind‐wandering may differ within richer, more semantically meaningful and vivid real‐world scenarios (Jordao et al. 2019). The impetus and freedom to explore the mind‐wandering space may unfold differently for younger and older adults in open‐ended, less circumscribed, natural environments (Maillet et al. 2018). Future research could incorporate open‐ended methods (see Irish et al. 2019) or MDES (e.g. Simola et al. 2023) to provide a further opportunity to gauge the richness of different inner mentations and processes as they evolve.

The strength of the current approach is in charting how strategic variation in attentional engagement as a function of age can influence discrete neural signals underpinning sensorimotor behaviour (i.e. sensory, motor, and decision formation signals). However, we recognise that a fuller picture of these dynamics must not only account for amplitude variation but also how changes in phase dynamics (e.g. the phase alignment of theta and alpha rhythms) can influence the precise timing of shifts between focused and mind‐wandering states (Hua et al. 2022). Further understanding of phase‐amplitude interactions will deepen our understanding of attentional fluctuations that characterise the exploitation/exploration tradeoff.

## 5 Conclusion

In summary, our research provides a new perspective on the influence of ageing on mind‐wandering, elucidating the distinct strategies employed by younger and older adults in line with the exploitation/exploration framework. We proffer that older adults suspend the wandering mind and implement a more exploitative oscillation strategy to allay potential costs and circumvent their reduced cognitive resources when the context demands it. This may represent an adaptive quality of successful ageing, namely, older adults prioritise task‐relevant information and choose their prime moment to explore. Conversely, younger adults exhibit greater exploration of the mind‐wandering space and utilise their greater cognitive resources to flexibly oscillate between competing goal‐directed and mind‐wandering strategies without incurring relative performance costs. Despite delayed evidence accumulation and reduced amplitude for target sensory evidence, younger adults show no corresponding decline in sustained attention performance, suggesting insulated mind‐wandering through perceptual decoupling.

## Conflicts of Interest

The authors declare no competing financial interests.

## IRB Statement

Participation was entirely voluntary, and all participants provided written informed consent to the procedures that were approved by the School of Psychology Research Ethics Committee, TCD, and were conducted in observance of the Declaration of Helsinki principles and the European General Data Protection Regulations.

## Data Availability

The data that support the findings of this study are available from the corresponding author upon reasonable request.
